# Novel brain-targeted nanomicelles for anti-glioma therapy mediated by the ApoE-enriched protein corona in vivo

**DOI:** 10.1186/s12951-021-01097-8

**Published:** 2021-12-28

**Authors:** Zhe-Ao Zhang, Xin Xin, Chao Liu, Yan-hong Liu, Hong-Xia Duan, Ling-ling Qi, Ying-Ying Zhang, He-ming Zhao, Li-Qing Chen, Ming-Ji Jin, Zhong-Gao Gao, Wei Huang

**Affiliations:** 1 State Key Laboratory of Bioactive Substance and Function of Natural Medicines, Institute of Materia Medica, Chinese Academy of Medical Sciences and Peking Union Medical College, Beijing, 100050 People’s Republic of China; 2 Beijing Key Laboratory of Drug Delivery Technology and Novel Formulations, Department of Pharmaceutics, Institute of Materia Medica, Chinese Academy of Medical Sciences and Peking Union Medical College, Beijing, 100050 China

**Keywords:** ApoE protein corona, Glioma, Targeting therapy, Paclitaxel

## Abstract

**Background:**

The interactions between nanoparticles (NPs) and plasma proteins form a protein corona around NPs after entering the biological environment, which provides new biological properties to NPs and mediates their interactions with cells and biological barriers. Given the inevitable interactions, we regard nanoparticle‒protein interactions as a tool for designing protein corona-mediated drug delivery systems. Herein, we demonstrate the successful application of protein corona-mediated brain-targeted nanomicelles in the treatment of glioma, loading them with paclitaxel (PTX), and decorating them with amyloid β-protein (Aβ)-CN peptide (PTX/Aβ-CN-PMs). Aβ-CN peptide, like the Aβ_1–42_ peptide, specifically binds to the lipid-binding domain of apolipoprotein E (ApoE) in vivo to form the ApoE-enriched protein corona surrounding Aβ-CN-PMs (ApoE/PTX/Aβ-CN-PMs). The receptor-binding domain of the ApoE then combines with low-density lipoprotein receptor (LDLr) and LDLr-related protein 1 receptor (LRP1r) expressed in the blood–brain barrier and glioma, effectively mediating brain-targeted delivery.

**Methods:**

PTX/Aβ-CN-PMs were prepared using a film hydration method with sonication, which was simple and feasible. The specific formation of the ApoE-enriched protein corona around nanoparticles was characterized by Western blotting analysis and LC–MS/MS. The in vitro physicochemical properties and in vivo anti-glioma effects of PTX/Aβ-CN-PMs were also well studied.

**Results:**

The average size and zeta potential of PTX/Aβ-CN-PMs and ApoE/PTX/Aβ-CN-PMs were 103.1 nm, 172.3 nm, 7.23 mV, and 0.715 mV, respectively. PTX was efficiently loaded into PTX/Aβ-CN-PMs, and the PTX release from rhApoE/PTX/Aβ-CN-PMs exhibited a sustained-release pattern in vitro. The formation of the ApoE-enriched protein corona significantly improved the cellular uptake of Aβ-CN-PMs on C6 cells and human umbilical vein endothelial cells (HUVECs) and enhanced permeability to the blood–brain tumor barrier in vitro. Meanwhile, PTX/Aβ-CN-PMs with ApoE-enriched protein corona had a greater ability to inhibit cell proliferation and induce cell apoptosis than taxol. Importantly, PTX/Aβ-CN-PMs exhibited better anti-glioma effects and tissue distribution profile with rapid accumulation in glioma tissues in vivo and prolonged median survival of glioma-bearing mice compared to those associated with PMs without the ApoE protein corona.

**Conclusions:**

The designed PTX/Aβ-CN-PMs exhibited significantly enhanced anti-glioma efficacy. Importantly, this study provided a strategy for the rational design of a protein corona-based brain-targeted drug delivery system. More crucially, we utilized the unfavorable side of the protein corona and converted it into an advantage to achieve brain-targeted drug delivery.

**Graphical Abstract:**

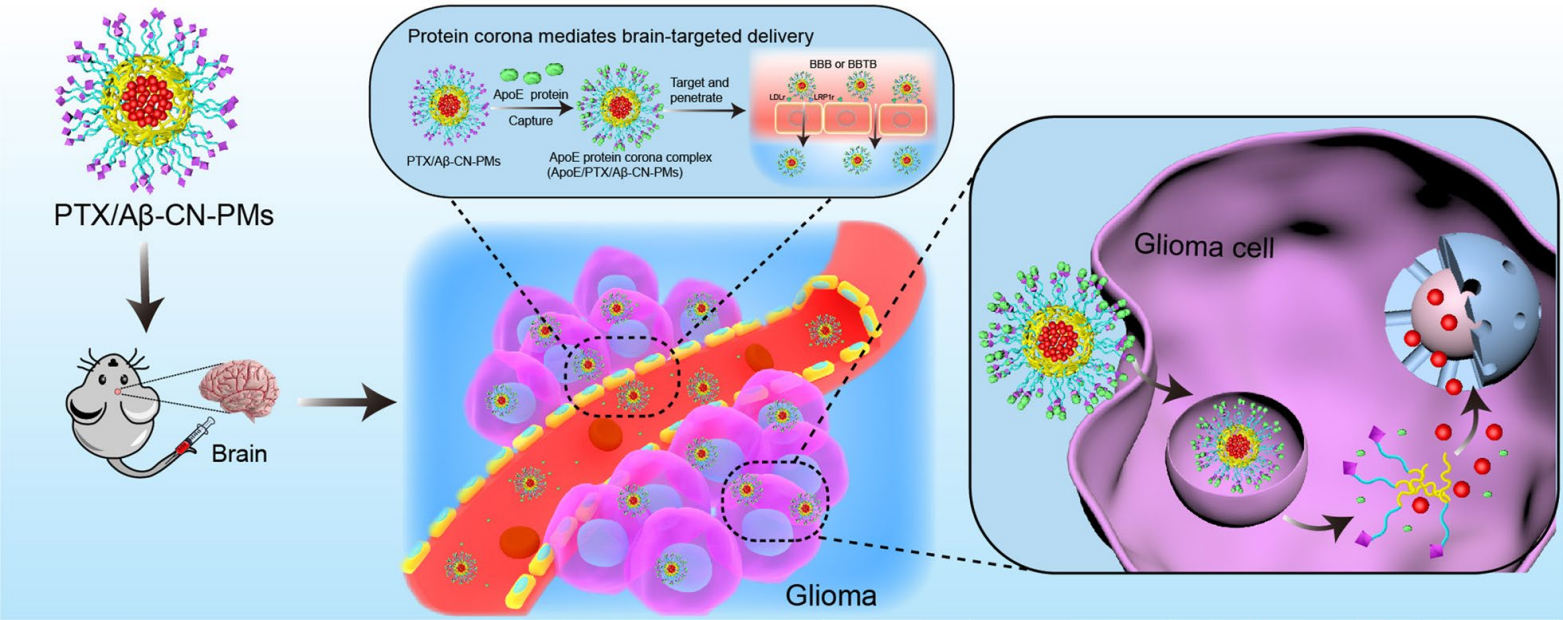

**Supplementary Information:**

The online version contains supplementary material available at 10.1186/s12951-021-01097-8.

## Background

Glioma, as the most common tumor of the central nervous system (CNS), is considered a fatal brain disease. According to the World Health Organization criteria, gliomas are classified into four groups: astrocytic tumors, oligodendrogliomas, ependymomas, and mixed gliomas. Glioblastoma (GBM) is the highest grade astrocytic tumor and is the most severe and aggressive type of primary brain tumor [1, 2]. The median survival time in patients with GBM is approximately 12–15 months, and approximately 5% of patients survive for up to 5 years [3, 4]. Surgery is currently the primary treatment for this disease; however, complete surgical resection of GBM is difficult because these tumors are frequently invasive, leading to disease progression or recurrence. Therefore, irradiation and chemotherapy play vital roles in the treatment of GBM [5, 6]. Because of the blood–brain barrier (BBB) and the blood–brain–tumor barrier (BBTB), most chemotherapeutic drugs are severely hindered from reaching the GBM region. To date, the efficacy of therapeutic agents through the BBB and BBTB remains a major challenge [7].

Nanocarriers are potential drug delivery systems for CNS therapeutic delivery. With the rapid development of nanotechnology, there has been considerable interest in utilizing nanocarriers to promote therapeutic efficacy and minimize the adverse effects of drugs. Due to the enhanced permeability and retention (EPR) effect, nanoparticles (NPs) are believed to preferentially accumulate in tumor regions. Although the brain-targeted delivery of nanomedicines depends on the EPR effect, active brain targeting via receptor-mediated transcytosis has been extensively explored to facilitate the targeted delivery of NPs to cross the BBB and BBTB because of their high specificity [8–10].

However, recent reports have indicated only mild improvements in active targeted delivery in the brain. Although NPs have been studied in a large number of preclinical and clinical trials, few NPs have been successfully translated for clinical use [11, 12]. When NPs enter biological fluids after systemic administration, they interact with biomolecules such as proteins to form protein corona (PC) [13, 14]. The formation of a PC changes the size, surface charge, aggregation state, and targeting properties of NPs, which ultimately determines the biological identity of NPs [15]. The biological identity of NPs plays an important role in the drug delivery, circulation time, cellular uptake, and cell recognition of NPs [16]. Nanoparticle–protein interactions and the formation of the PC are major challenges for drug delivery. Meanwhile, the interactions and PC may be used to decorate NPs and adjust the type of PC to enhance the targeting efficiency [17, 18]. Therefore, by regulating the interactions between NPs and biological systems in situ, the surface-adsorbed PC provides insights into the use of the PC as a targeting agent for drug delivery and effective biomedical applications of NPs.

Apolipoprotein E (ApoE), an endogenous protein (40–70 μg/mL), is composed of 299 amino acid residues and has a molecular weight of approximately 36 kDa. The structure of ApoE segregates into two ordered segments connected by residues 165–200, which are random segments [19, 20]. ApoE has many important functions. First, the amino-terminal domain of ApoE consists of a four-helix bundle. Its low-density lipoprotein (LDL) receptor-binding region containing residues 136–150 is within this four-helix bundle and includes rich lysyl and arginyl residues; therefore, drug delivery may be mediated through the interactions of ApoE with the LDL receptor (LDLr) and LDLr-related protein 1 receptor (LRP1r) on cancer cells. Second, the lipid-binding site involves residues 244–272 of the C-terminal domain of ApoE and has a high percentage of α-helices [21, 22]. ApoE, regarded as an exchangeable apolipoprotein, participates in the transport of lipids between the brain and plasma.

The 11-amino acid fragment amyloid β-protein (Aβ)_25–35_ is widely considered as a substitute for the full-length peptide Aβ_1–42_. The toxic properties of Aβ_25–35_ are changed by converting the C-terminal carboxylate into an amide, called the Aβ-CN peptide in the present study [23]. The carboxy-terminal lipid-binding domain of ApoE reportedly mediates the binding activity of ApoE with Aβ while inhibiting the binding of the C-terminal domain of ApoE with other lipids due to the formation of Aβ/ApoE complexes. Aβ/ApoE complexes can penetrate the BBB because the receptor-binding site of ApoE effectively binds to the LDLr and LRP1r expressed on the BBB and glioma in the proper conformation [24–26].

**Scheme 1:**
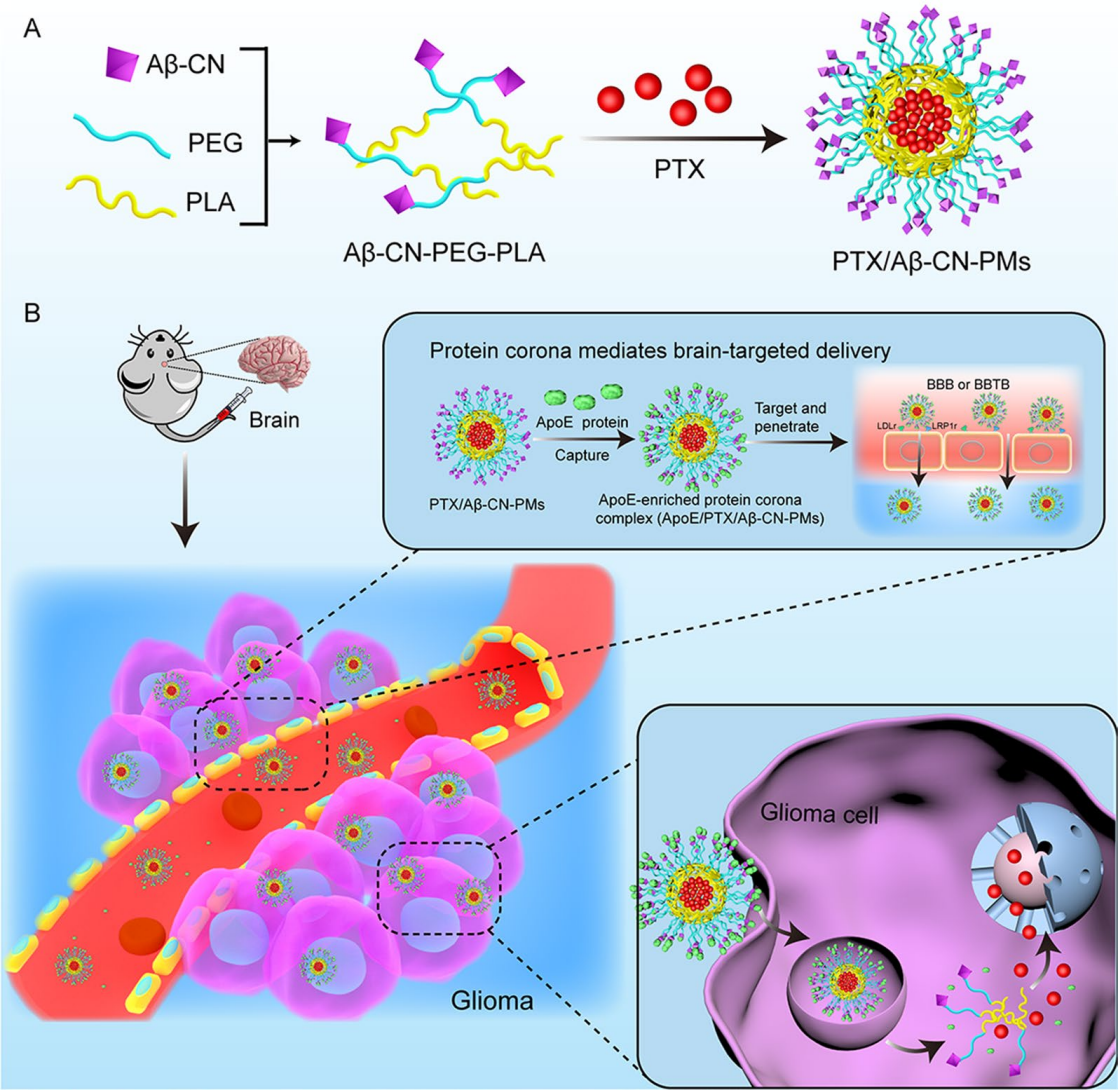
**A** PTX loaded Aβ-CN-PMs (PTX/Aβ-CN-PMs) were prepared by film-hydration method with sonication. **B** PTX/Aβ-CN-PMs effectively captured ApoE and the brain targeted ApoE protein corona surrounding PTX/Aβ-CN-PMs crossed the BBB and BBTB, following by endocytosis into glioma cells.

Hence, in the present study, Aβ_25–35_ with an amide bond (Aβ-CN) was decorated on poly(ethylene glycol) (PEG)–poly(lactic acid) (PLA) nanomicelles (PMs) and was used to bind the C-terminal domain of ApoE upon exposure to physiological environments. ApoE-enriched PC then forms in situ on the surface of Aβ-CN-PMs. The ApoE-enriched PC specifically targets the BBB and glioma cells via LDLr- and LRP1r-mediated endocytosis (Scheme 1). The PTX/Aβ-CN-PMs were prepared and optimized, and their physicochemical properties were characterized. The in vitro and in vivo antitumor efficacy of PTX/Aβ-CN-PMs was also evaluated. We discuss the potential of the ApoE-enriched PC as a targeting strategy for brain delivery, which provides novel insights into glioma therapy and further improves the design of targeted drug delivery systems with high efficiency and enhanced safety for better clinical translation.

## Results and discussion

### Synthesis and characterization of mPEG–PLA and Mal-PEG–PLA

**Fig. 1:**
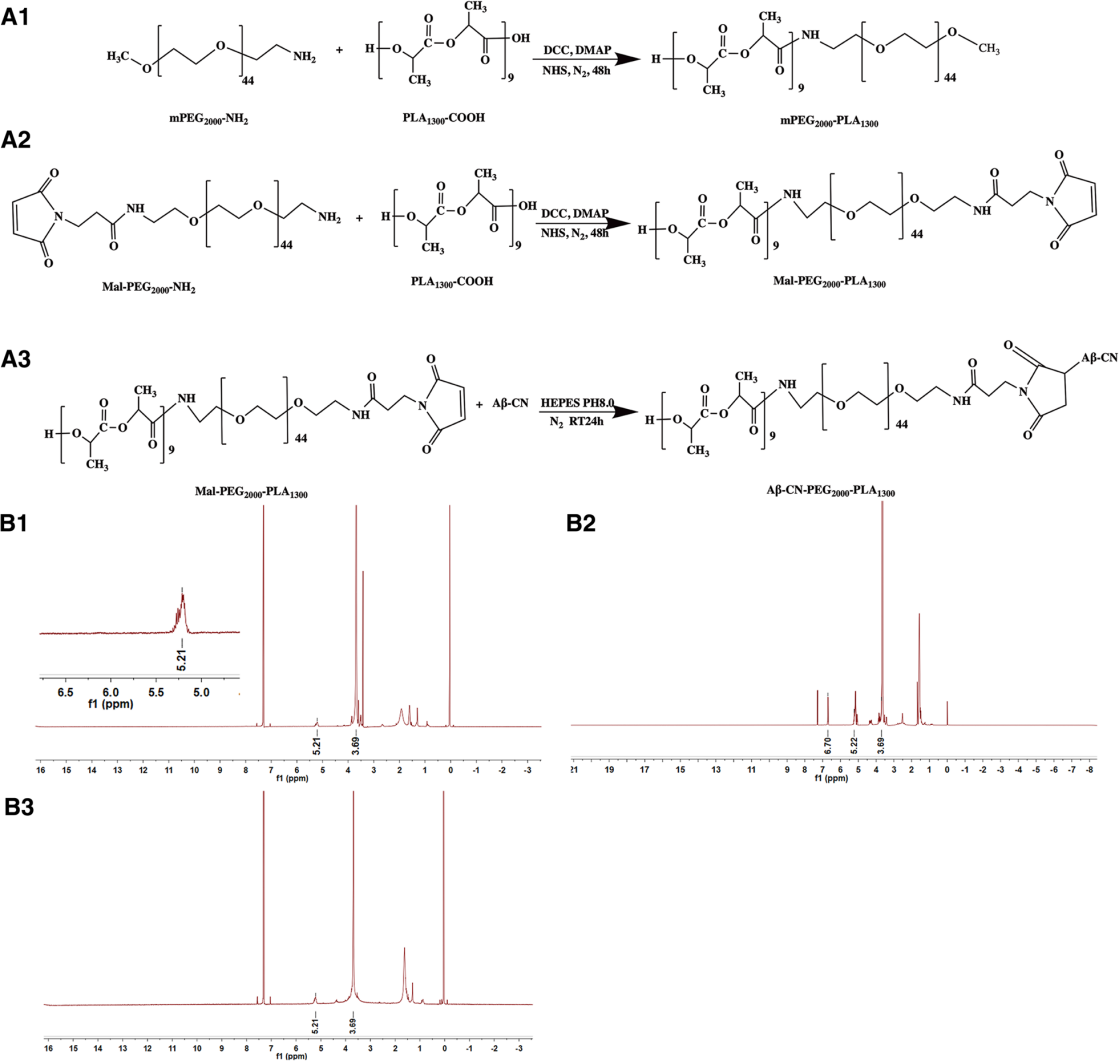
Synthesis and characterization of mPEG_2000_-PLA_1300_, Mal-PEG_2000_-PLA_1300_ and Aβ-CN-PEG_2000_-PLA_1300_. **A** Synthesis route of mPEG_2000_-PLA_1300_ (**A1**), Mal- PEG_2000_-PLA_1300_ (**A2**), and Aβ-CN-PEG_2000_-PLA_1300_ (**A3**). **B** ^1^H-NMR spectra of mPEG_2000_-PLA_1300_ (**B1**), Mal-PEG_2000_-PLA_1300_ (**B2**), and Aβ-CN-PEG_2000_-PLA_1300_ (**B3**)

The synthetic routes of mPEG_2000_–PLA_1300_ and Mal-PEG_2000_–PLA_1300_ are shown in Fig. 1A1, A2. mPEG_2000_–PLA_1300_ and Mal-PEG_2000_–PLA_1300_ were synthesized via an amidation reaction of the carboxyl group of PLA_1300_-COOH and amine of mPEG_2000_-NH_2_ and Mal-PEG_2000_-NH_2_, respectively. The ^1^H-nuclear magnetic resonance (^1^H-NMR) spectra of mPEG_2000_–PLA_1300_ in CDCl_3_ was shown in Fig. 1B1. The chemical shifts at 3.69 ppm and 5.21 ppm were separately referred to as the characteristic resonance of the PEG_2000_ segment (–OCH_2_–CH_2_–) and PLA_1300_ segment (–CH–), respectively. The ^1^H-NMR spectra of Mal-PEG_2000_–PLA_1300_ was presented in Fig. 1B2. The signature resonance peaks of the PEG_2000_ segment (–OCH_2_–CH_2_–) and PLA_1300_ segment (–CH–) appeared at chemical shifts at 3.69 ppm and 5.22 ppm, respectively, which was similar to the mPEG_2000_–PLA_1300_ spectra. In addition, the peak at 6.70 ppm was the characteristic peak of the maleimide group of Mal-PEG_2000_–PLA_1300_. These results confirmed the successful conjugation of mPEG_2000_–PLA_1300_ and Mal-PEG_2000_–PLA_1300_, which were used for further study.

### Synthesis and characterization of Aβ-CN-PEG–PLA

To prepare functional polymer materials, the Aβ-CN peptide with the sequence NH_2_-CGSNKGAIIGLM-CONH was conjugated to Mal-PEG_2000_–PLA_1300_ using a sulfhydryl-maleimide coupling method, as previously reported. Aβ-CN-PEG_2000_–PLA_1300_ was prepared, as shown in Fig. 1A3. The structure of Aβ-CN-PEG_2000_–PLA_1300_ copolymer was confirmed via ^1^H-NMR analysis. Fig. 1B3 shows that the characteristic signal of the maleimide group of Mal-PEG_2000_–PLA_1300_ at 6.70 ppm disappeared, indicating that the maleimide group completely reacted with the thiol group of the Aβ-CN peptide.

### Preparation and characterization of PTX-loaded Aβ-CN-PEG–PLA micelles

**Fig. 2:**
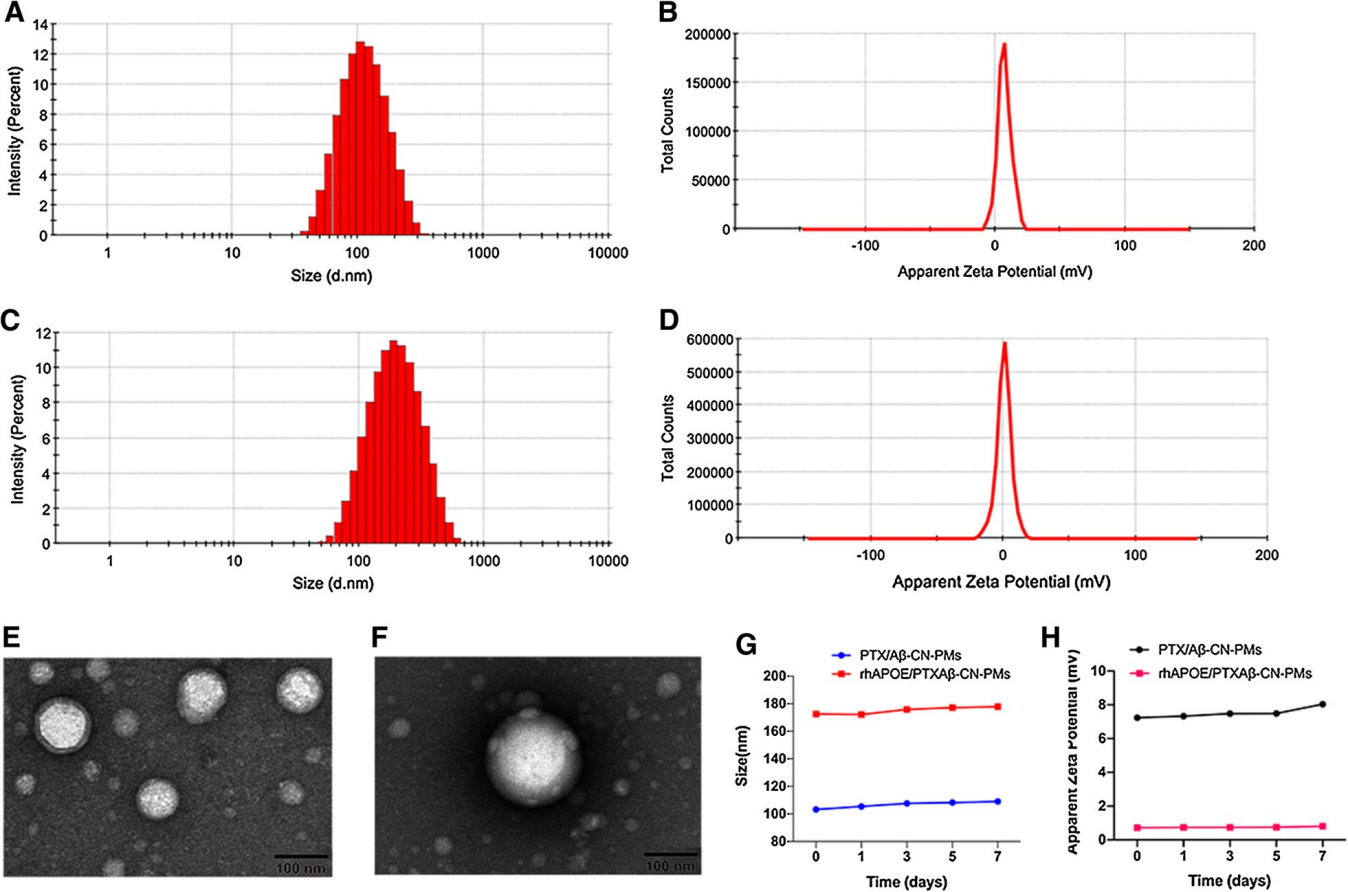
Characterizations of PTX/Aβ-CN-PMs and ApoE/PTX/Aβ-CN-PMs. **A**, **C** Size distribution of PTX/Aβ-CN-PMs and ApoE/PTX/Aβ-CN-PMs by dynamic light-scattering analysis. **B**, **D** Zeta potential distribution of PTX/Aβ-CN-PMs and ApoE/PTX/Aβ-CN-PMs. **E**, **F** TEM images of PTX/Aβ-CN-PMs and ApoE/PTX/Aβ-CN-PMs; scale bar = 100 nm. **G** The size stability of PTX/Aβ-CN-PMs and ApoE/PTX/Aβ-CN-PMs for seven days. **H** The zeta potential stability of PTX/Aβ-CN-PMs and ApoE/PTX/Aβ-CN-PMs for seven days

PEG–PLA micelles have been extensively used to deliver various hydrophobic therapeutic drugs because of their safety and biocompatibility. PTX-loaded Aβ-CN-PEG_2000_–PLA_1300_ micelles (PTX/Aβ-CN-PMs) were prepared using a film hydration method with sonication. The PTX/Aβ-CN-PMs were characterized for particle size, size distribution, zeta potential, and stability. As shown in Fig. 2A and B, the average hydrodynamic diameter of PTX/Aβ-CN-PMs was 103.1 nm, and its polydispersity index was 0.184, with a narrow distribution. The zeta potential of the PTX/Aβ-CN-PMs was 7.23 mV, indicating that PTX/Aβ-CN-PMs had a positive zeta potential to facilitatly adsorb the apolipoproteins with negative charge and form the ApoE-enriched protein corona. The PTX/Aβ-CN-PMs exhibited good stability with very minor changes of size and zeta potential in phosphate-buffered saline (PBS) during seven days at room temperature (Fig. 2G, H). The morphology of PTX/Aβ-CN-PMs was observed using transmission electron microscopy (TEM), and the TEM images in Fig. 2E indicated that the PTX/Aβ-CN-PMs were spherical with a smooth surface. The encapsulation efficiency (EE%) and loading capacity (LC%) of the PTX/Aβ-CN-PMs were 90.3% and 8.25%, respectively. These data suggested that PTX was effectively loaded and that Aβ-CN-PMs were an excellent brain delivery nanocarrier for PTX.

### Interaction and analysis of ApoE on Aβ-CN-PEG–PLA micelles in vitro

**Fig. 3:**
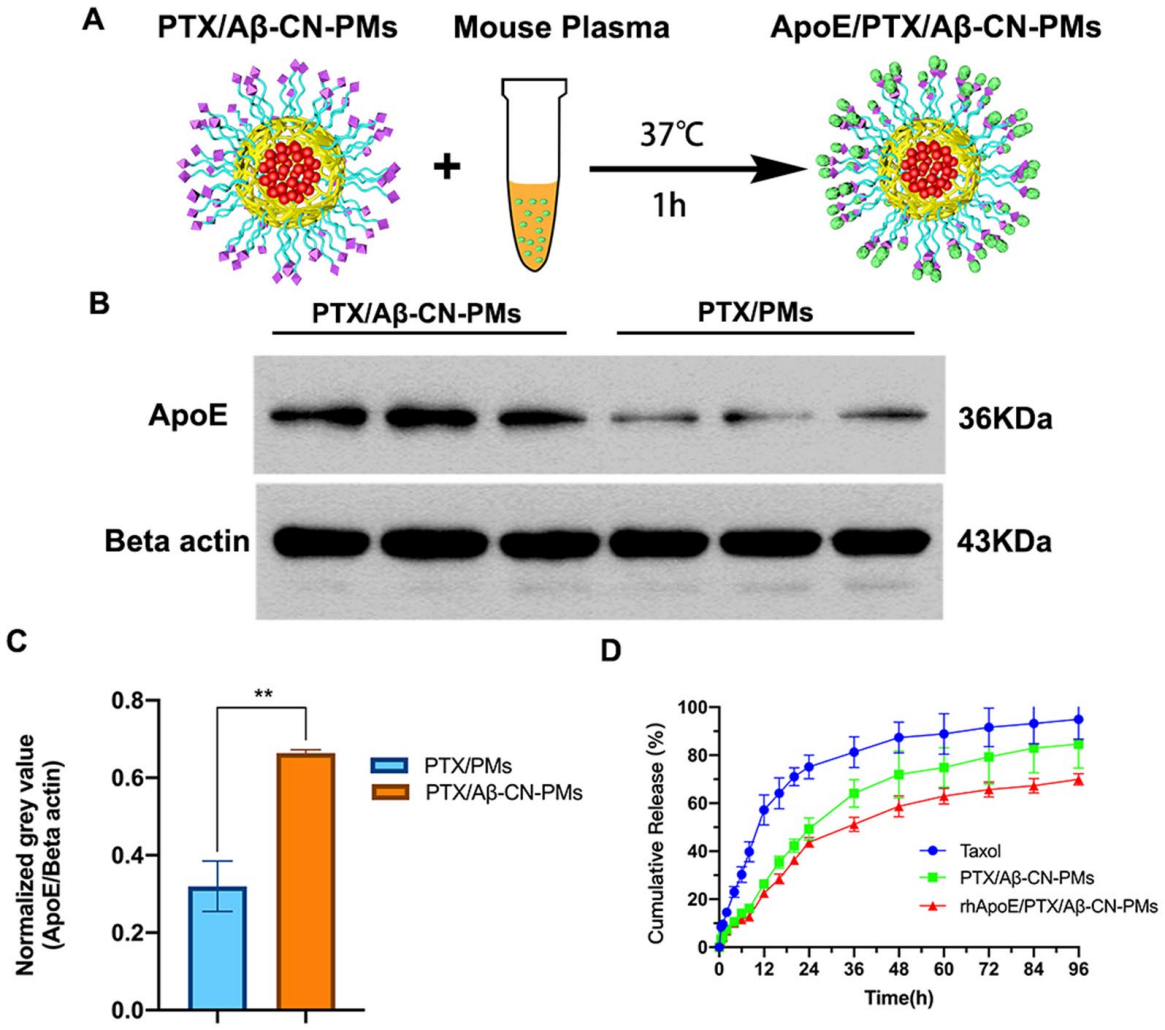
**A** Schematic diagram of PTX/Aβ-CN-PMs were incubated with the mouse plasma for 1 h at 37 °C in vitro. **B** Western Blotting results of the absorbed ApoE on PTX/PMs and PTX/Aβ-CN-PMs. **C** Gel Image system ver.4.00 (Tanon, China) was performed to quantitatively analyze the absorbed protein ApoE. means ± SD, n = 3; *P < 0.05, **P < 0.01, ***P < 0.001. **D** The PTX release profiles of taxol, PTX/Aβ-CN-PMs and rhApoE/PTX/Aβ-CN-PMs in sodium salicylate solution (1 M, pH 7.4)

To further study the formation of ApoE-enriched protein corona on the surface of PTX/Aβ-CN-PMs, PTX/Aβ-CN-PMs and PTX/PMs were incubated with mouse plasma for 1 h at 37 °C under continuous agitation in vitro (Fig. 3A). After incubation, the PMs absorbed ApoE-enriched protein corona were collected via centrifugation and suspended in cold PBS thrice, as described in the Methods section. The content of ApoE in the formed PC in vitro was then confirmed and analyzed via western blotting analysis. As shown in Fig. 3B, C, protein bands identified as ApoE (36 kDa) were found on the PC of PTX/Aβ-CN-PMs and PTX/PMs. However, the normalized gray value of ApoE absorbed on the PTX/Aβ-CN-PMs (ApoE/PTX/Aβ-CN-PMs) was twofold higher than the ApoE absorbed on the PTX/PMs. These results indicated that the concentration of ApoE in the PC of PTX/Aβ-CN-PMs was higher as a result of Aβ-CN peptide modification and that Aβ-CN-PMs increased the specific capturing of brain-targeting ApoE.

To further confirm the nanoparticle–protein interactions, the physical characterizations of ApoE/PTX/Aβ-CN-PMs such as size, zeta potential and stability were investigated. Figure 2C, D revealed that the hydrodynamic size and zeta potential of ApoE/PTX/Aβ-CN-PMs was 172.3 nm, with a narrow distribution, and 0.715 mV. The TEM image in Fig. 2F showed a layer surrounding the PTX/Aβ-CN-PMs, which indicated the formation of protein corona. No significant size increase and zeta potential changes was found after incubation with plasma for seven days, demonstrating the high stability of the ApoE/PTX/Aβ-CN-PMs (Fig. 2G, H).

Therefore, to simulate the in vivo adsorption of ApoE and Aβ-CN-PMs, recombinant human (rh)ApoE and PTX/Aβ-CN-PMs were incubated in vitro for 1 h at 37 °C with gentle shaking. The Aβ-CN-PMs bound to rhApoE (rhApoE/PTX/Aβ-CN-PMs) were then separated and obtained via centrifugation (14,000 rpm, 1 h) as described above for subsequent in vitro experiments.

### Characterization of protein corona

**Fig. 4:**
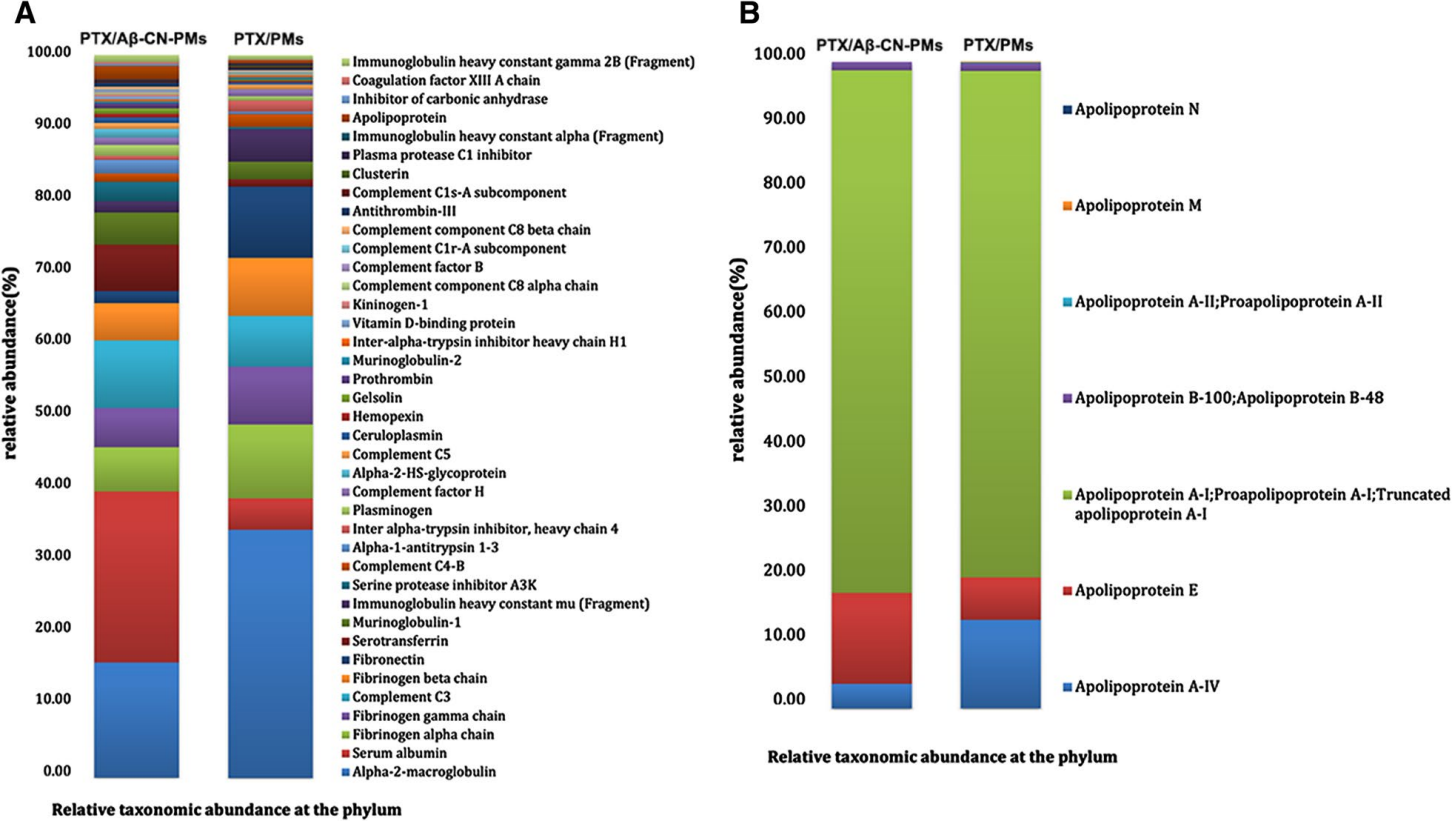
Protein corona characterization. **A** The types and abundance of the adsorbed proteins on PTX/Aβ-CN-PMs and PTX/PMs. **B** The types and abundance of apolipoproteins on PTX/Aβ-CN-PMs and PTX/PMs

A comprehensive identification of proteins associated with PTX loaded PMs following incubation of PTX/Aβ-CN-PMs and PTX/PMs with mouse plasma was performed by LC–MS/MS. Figure 4A shows the types and abundance of the adsorbed proteins on PTX/Aβ-CN-PMs and PTX/PMs. The 40 most abundant proteins were identified and ranked. The amount of serum albumin, fibrinogen and complement on the protein corona in the PTX/Aβ-CN-PMs and PTX/PMs group was higher compared to other proteins on the protein corona. Surprisingly, it was also noted that the relative quantity of apolipoproteins absorbed on PTX/Aβ-CN-PMs increased with respect to PTX/PMs. The weight percentage of apolipoproteins in the PTX/Aβ-CN-PMs group was 1.87%, which was 5.02-fold higher than that of PTX/PMs group. Importantly, the weight percentage of ApoE in the apolipoproteins absorbed on the surface of PTX/Aβ-CN-PMs was 13.3%, twofold higher than that of absorbed on the PTX/PMs (Fig. 4B). Thus, the weight percentage of ApoE was 0.247% in the PTX/Aβ-CN-PMs group. In contrast, the weight percentage of ApoE in the PTX/PMs group was 0.024%. The above results suggested that the Aβ-CN peptide modification of PTX/Aβ-CN-PMs improved the amount of ApoE in protein corona in comparison to PTX/PMs.

### In vitro release study

The release profiles of taxol, PTX/Aβ-CN-PMs, and rhApoE/PTX/Aβ-CN-PMs were characterized in the release media. The accumulation drug release curves are shown in Fig. 3D. Within 96 h, the cumulative release values of PTX from taxol, PTX/Aβ-CN-PMs, and rhApoE/PTX/Aβ-CN-PMs were 94.92%, 84.56%, and 69.92%, respectively. The release of PTX from taxol and PTX/Aβ-CN-PMs was rapid, where approximately 80% and 71.99% of the PTX in these formulations were released into the media within 48 h, respectively. The PTX release value from the rhApoE/PTX/Aβ-CN-PMs was 58.73%, showing sustained drug release without a burst effect. This suggested that the formation of the ApoE-enriched protein corona facilitated the slow release of drugs, enabling PTX to accumulate in tumor tissues effectively.

### In vitro cytotoxicity

**Fig. 5:**
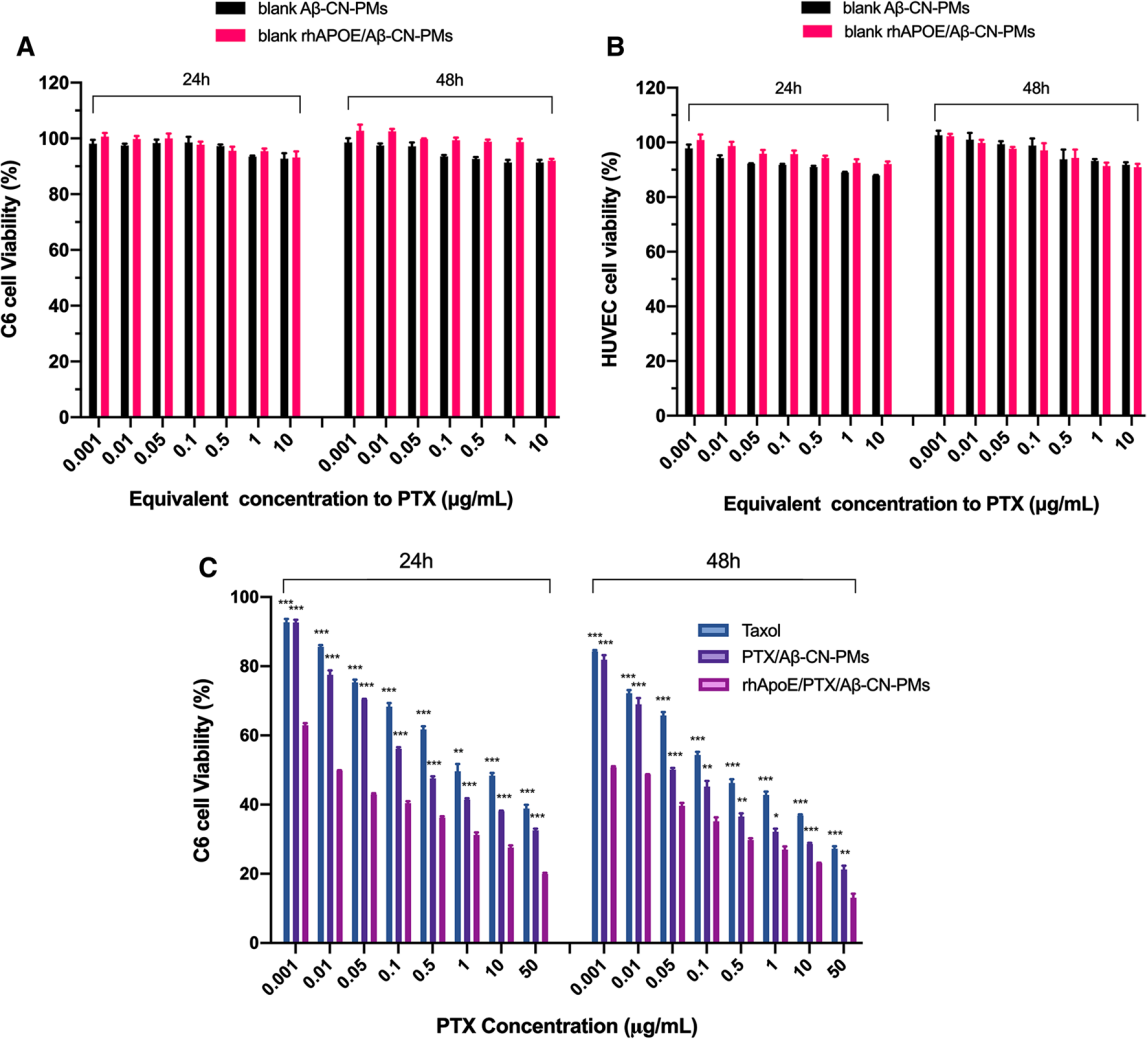
In vitro cytotoxicity study. In vitro cytotoxicity of blank Aβ-CN-PMs and rhApoE/Aβ-CN-PMs on C6 cells (**A**) and on HUVEC cells (**B**). **C** Inhibitory capacity of Taxol, PTX/Aβ-CN-PMs and rhApoE/PTX/Aβ-CN-PMs against C6 cells proliferation. Means ± SD, n = 3; *P < 0.05, **P < 0.01, ***P < 0.001 versus the rhApoE/PTX/Aβ-CN-PMs group

The cell viability of C6 cells and HUVECs after incubation with blank Aβ-CN-PMs and rhApoE/Aβ-CN-PMs are shown in Fig. 5A, B. The cell viability of C6 cells and HUVECs were higher than 85% with the equivalent PTX concentration ranging from 0.001 to 10 µg/mL after 24 h or 48 h incubation with blank Aβ-CN-PMs and rhApoE/Aβ-CN-PMs. These results suggested that Aβ-CN-PMs as drug delivery nanocarriers showed excellent safety.

The effects of taxol, PTX/Aβ-CN-PMs, and rhApoE/PTX/Aβ-CN-PMs on the proliferation of C6 cells were also investigated using the Cell Counting Kit-8 (CCK-8) assay. The data shown in Fig. 5C indicated that rhApoE/PTX/Aβ-CN-PMs effectively demonstrated inhibitory capacity at PTX concentrations ranging from 0.001 to 10 µg/mL compared with taxol and PTX/Aβ-CN-PMs in a dose- and time-dependent manner. The IC_50_ values of taxol were 0.9781 μg/mL and 0.2771 μg/mL for 24 h and 48 h incubation, respectively, while they were only 0.0369 μg/mL and 0.02157 μg/mL, respectively, for the rhApoE/PTX/Aβ-CN-PMs. These results showed that rhApoE/PTX/Aβ-CN-PMs had a better inhibitory capacity than taxol and PTX/Aβ-CN-PMs, which might be attributed to the increased uptake on C6 cells due to the absorption of the brain-targeting protein ApoE.

### In vitro cellular uptake

**Fig. 6:**
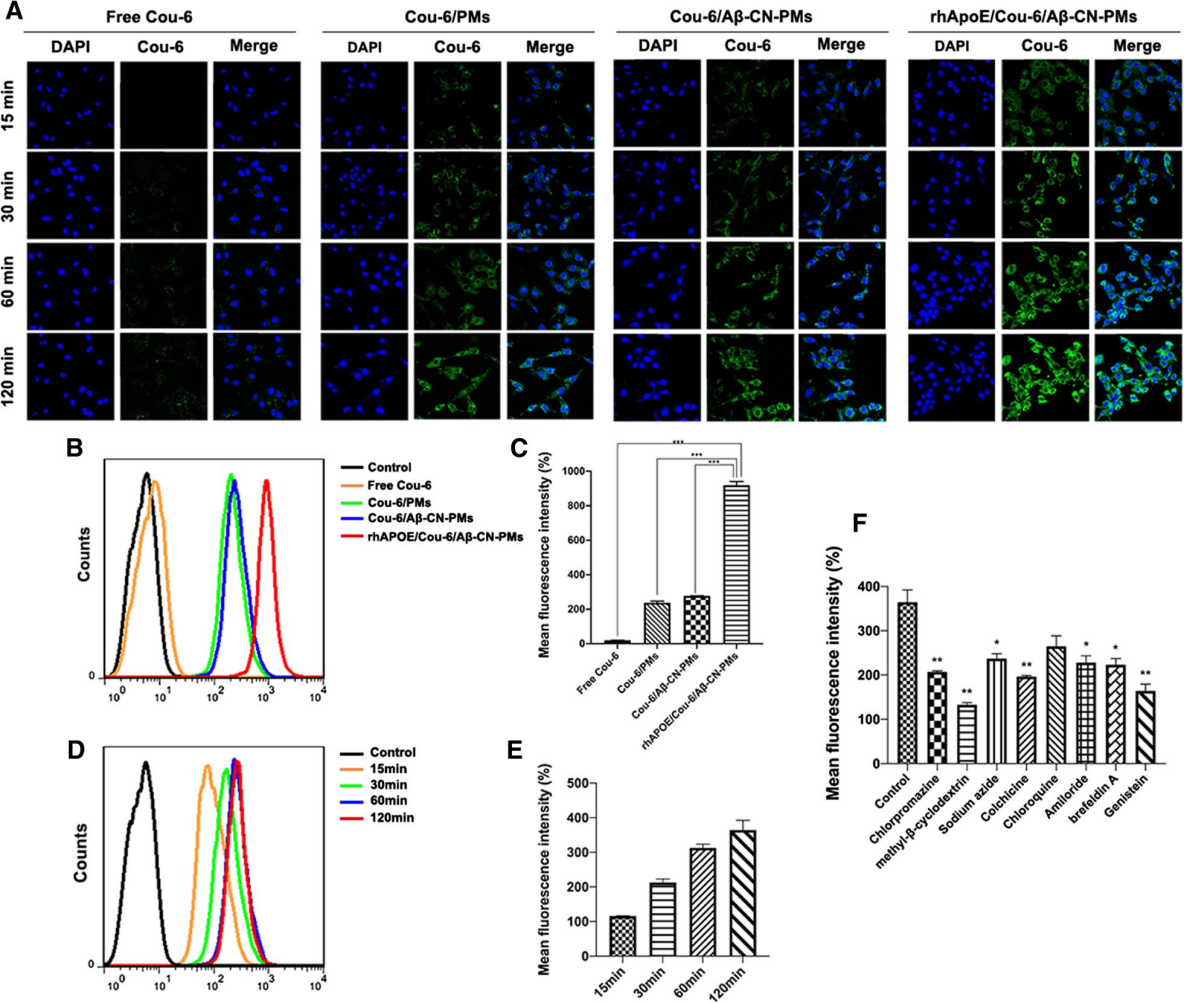
In vitro cellular uptake on C6 cells. **A** CLSM observation of C6 cells after treatment with free Cou-6, Cou-6/PMs, Cou-6/Aβ-CN-PMs, rhApoE/Cou-6/Aβ-CN-PMs for 15, 30, 60 and 120 min (magnification × 200), respectively. **B**, **C** Quantitative analysis of cellular uptake by flow cytometry after incubation with free Cou-6, Cou-6/PMs, Cou-6/Aβ-CN-PMs, rhApoE/Cou-6/Aβ-CN-PMs for 2 h, respectively. Means ± SD, n = 3; *P < 0.05, **P < 0.01, ***P < 0.001 versus rhApoE/Cou-6/Aβ-CN-PMs group. **D**, **E** Quantitative analysis of cellular uptake by flow cytometry after incubation with rhApoE/Cou-6/Aβ-CN-PMs for 15, 30, 60 and 120 min. **F** Cellular uptake analysis of rhApoE/Cou-6/Aβ-CN-PMs after incubation with different endocytic inhibitors by flow cytometry. Means ± SD, n = 3; *P < 0.05, **P < 0.01, ***P < 0.001 versus the control group without endocytic inhibitors

**Fig. 7:**
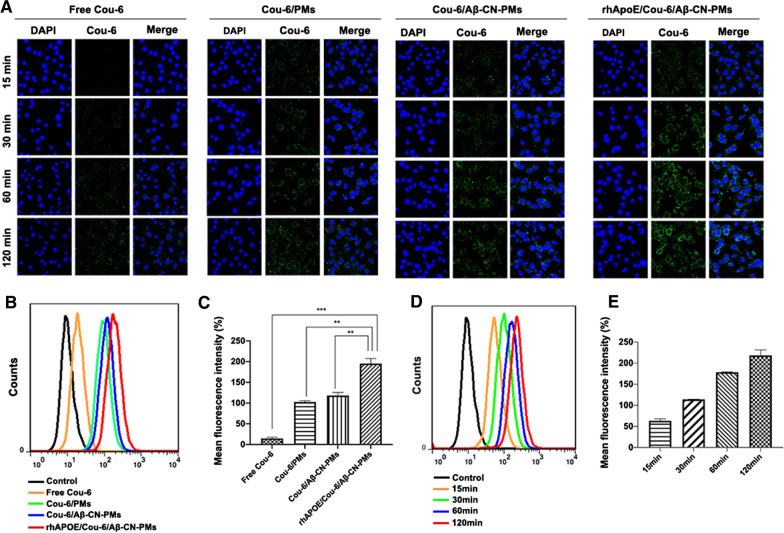
In vitro cellular uptake on HUVEC cells. **A** CLSM observation of HUVEC cells after treatment with free Cou-6, Cou-6/PMs, Cou-6/Aβ-CN-PMs, rhApoE/Cou-6/Aβ-CN-PMs for 15, 30, 60 and 120 min(magnification × 200), respectively. **B**, **C** Quantitative analysis of cellular uptake by flow cytometry after incubation with free Cou-6, Cou-6/PMs, Cou-6/Aβ-CN-PMs, rhApoE/Cou-6/Aβ-CN-PMs for 2 h, respectively. **D**, **E** Quantitative analysis of cellular uptake by flow cytometry after incubation with rhApoE/Cou-6/Aβ-CN-PMs for 15, 30, 60 and 120 min. Means ± SD, n = 3; *P < 0.05, **P < 0.01, ***P < 0.001 versus rhApoE/Cou-6/Aβ-CN-PMs group

The cellular uptake of the different formulations was determined qualitatively and quantitatively on C6 cells and HUVECs. Coumarin-6 (Cou-6) was incorporated into different types of PMs as a fluorescence indicator. On C6 cells and HUVECs, as shown in Figs. 6B, C, 7B, C, flow cytometry results indicated that the uptake of Cou-6 in the rhApoE/Cou-6/Aβ-CN-PMs group was more substantial than that of Cou-6 in other groups after 2 h of incubation. The uptake of rhApoE/Cou-6/Aβ-CN-PMs on C6 cells and HUVECs revealed a time-dependent pattern, reaching saturation after 2 h of incubation (Figs. 6D, E, 7D, E). A similar phenomenon was also observed in the confocal fluorescence microscopy (CLSM) results shown in Figs. 6A and 7A, wherein the fluorescence intensity of Cou-6 in the rhApoE/Cou-6/Aβ-CN-PMs group was higher than that of other groups at different time intervals and exhibited the strongest fluorescence signal at 2 h. These results suggested that the ApoE-enriched PC promoted the rapid uptake of rhApoE/Cou-6/Aβ-CN-PMs on C6 cells and HUVECs, further speculating that the formation of the in situ brain-targeting ApoE-enriched PC in vivo is beneficial for crossing the BBB to target glioma cells and to achieve the efficient intracerebral delivery of chemotherapeutics. In addition, the cellular uptake of Cou-6/PMs on C6 cells and HUVECs was similar to that of Cou-6/Aβ-CN-PMs, indicating that PMs without the formation of the ApoE-enriched PC showed little capacity to increase the cellular uptake of C6 cells and HUVECs. Therefore, the ApoE-enriched PC may be the key to enhancing brain-targeting efficiency.

Different inhibitors were used to investigate the possible endocytic pathways of rhApoE/Cou-6/Aβ-CN-PMs using flow cytometry. As shown in Fig. 6F, methyl-β-cyclodextrin (M-β-CD), an inhibitor of lipid raft-mediated endocytosis that acts by reversibly extracting steroids out of the plasma membrane [37], was critical for the uptake of rhApoE/Cou-6/Aβ-CN-PMs on C6 cells. After C6 cells were treated with M-β-CD, the cellular uptake of rhApoE/Cou-6/Aβ-CN-PMs decreased by approximately 59%, suggesting that lipid raft-mediated endocytosis is the dominant cellular uptake mechanism of rhApoE/Cou-6/Aβ-CN-PMs. Meanwhile, caveolae-mediated endocytosis, which is typically inhibited by genistein, a tyrosine kinase inhibitor, played a major role in the uptake of rhApoE/Cou-6/Aβ-CN-PMs [38]. The fluorescence intensity of Cou-6 was reduced by 49% when the cells were treated with genistein. Therefore, these results indicated that the cellular uptake of rhApoE/Cou-6/Aβ-CN-PMs depended significantly on lipid raft-mediated and caveolae-mediated endocytosis. Consistent with previous reports, this finding reflected a well-characterized caveola/raft-dependent endocytotic pathway [39]. In this uptake mechanism, the extracellular complexes combine with the “caveola” domains on the cell membrane, which are associated with high cholesterol levels. Subsequently, the activation of tyrosine kinases causes a signaling cascade that ultimately leads to the effective internalization of a substance [40].

In addition, cells treated with chlorpromazine (an inhibitor of clathrin-mediated endocytosis), colchicine (an inhibitor of microtubule formation and macropinocytosis), and brefeldin A (an inhibitor of protein transport from the endoplasmic reticulum to the Golgi apparatus) showed a 36%, 39%, and 31% decrease in cellular uptake, respectively as reflected in the decrease in fluorescence signals. The cellular uptake of rhApoE/Cou-6/Aβ-CN-PMs was partially dependent on clathrin-mediated endocytosis, macropinocytosis, and the Golgi apparatus [41–43]. Sodium azide (NaN_3_) is used to inhibit energy-dependent endocytosis by disrupting ATP production. The intracellular fluorescence intensity of cells treated with NaN_3_ decreased by approximately 26% compared to the control group, confirming that the cellular uptake of rhApoE/Cou-6/Aβ-CN-PMs was moderately energy-dependent [44, 45].

Based on these results, it was clear that not only the endocytic pathway was involved in rhApoE/Cou-6/Aβ-CN-PMs uptake. The lipid raft-mediated and caveolae-mediated endocytosis pathways were the main mechanisms by which rhApoE/Cou-6/Aβ-CN-PMs entered cells in an energy-dependent manner. In contrast, clathrin-mediated endocytosis, macropinocytosis, and the Golgi apparatus contributed less to the cellular uptake of rhApoE/Cou-6/Aβ-CN-PMs.

### Cell apoptosis assay

**Fig. 8:**
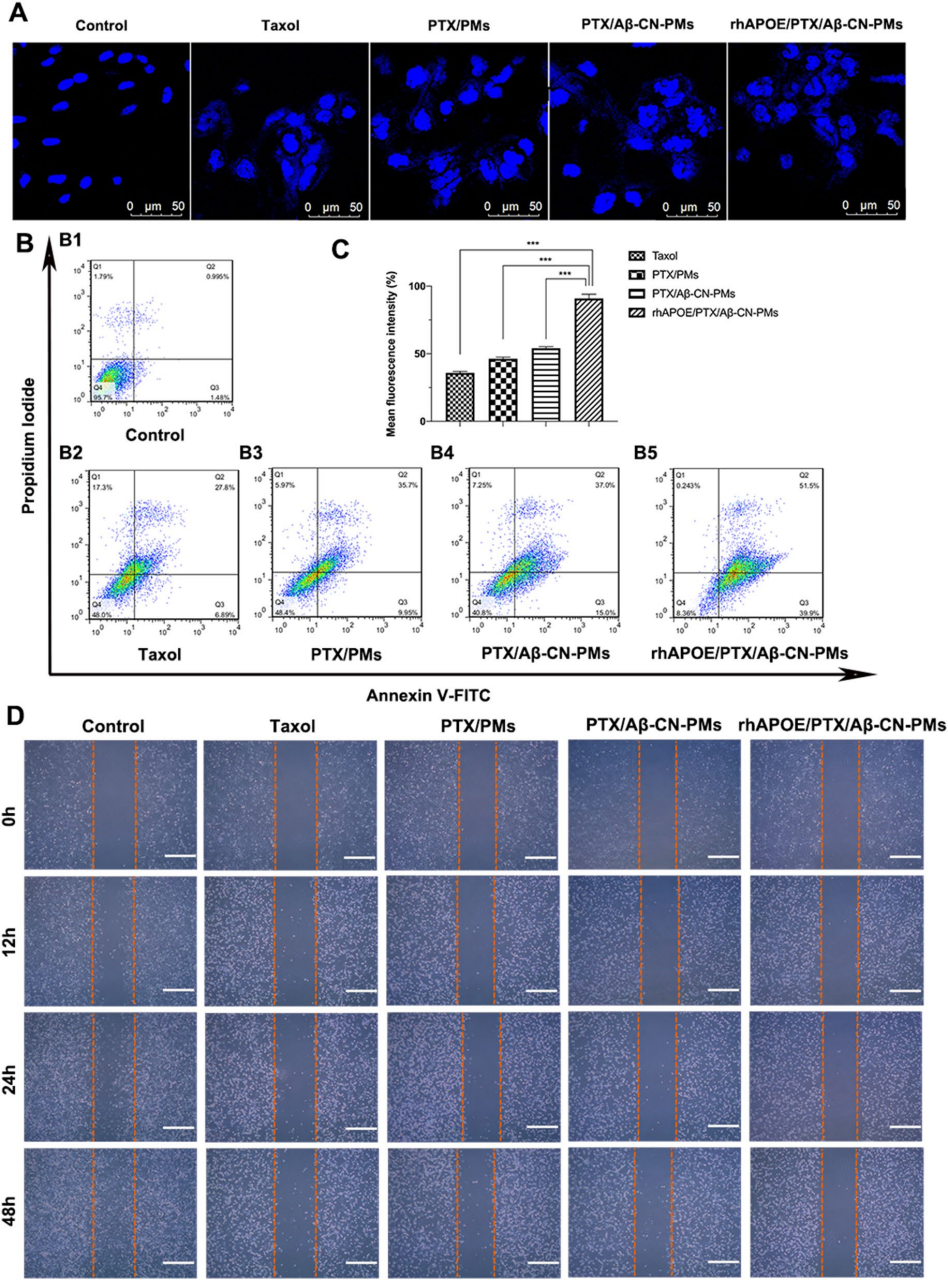
Cell apoptosis and cell wound healing assay on C6 cells in vitro. **A** The CLSM images of C6 cells with Hoechst staining after the 48 h incubation with taxol, PTX/PMs, PTX/Aβ-CN-PMs and rhApoE/PTX/Aβ-CN-PMs (magnification × 200). Scale bar = 50 μm. **B**, **C** The flow cytometry results of C6 cell apoptosis and the percentage of early and late apoptosis after the 48 h treatment with Taxol, PTX/PMs, PTX/Aβ-CN-PMs and rhApoE/PTX/Aβ-CN-PMs (n = 3, Means ± SD), *P < 0.05, **P < 0.01, ***P < 0.001. **D** Wound-healing assay on C6 cells and the images were captured at 0 h, 12 h, 24 h and 48 h (magnification × 100), scale bar = 100 μm

CLSM and flow cytometry were used to evaluate C6 cell apoptosis induced by taxol, PTX/PMs, PTX/Aβ-CN-PMs, and rhApoE/PTX/Aβ-CN-PMs (PTX concentration of 1 μg/mL), with complete culture medium as the control. As shown in Fig. 8A, the nuclei of C6 cells in the control group were spherical and integral with homogeneous fluorescence after Hoechst 33258 staining. The nuclei of cells incubated with taxol, PTX/PMs, and PTX/Aβ-CN-PMs were moderately damaged. The nuclei of C6 cells were severely fragmented when exposed to rhApoE/PTX/Aβ-CN-PMs, suggesting that the nuclei were condensed and distributed into apoptotic bodies. Additionally, similar quantitative results were observed using flow cytometry. Figure 8B and C show that the percentage of cells in the early and late apoptosis stages was 90.9% after 48 h incubation with rhApoE/PTX/Aβ-CN-PMs, which was significantly higher than 35.8%, 46.4%, and 54.1% for taxol, PTX/PMs, and PTX/Aβ-CN-PMs, respectively. These results indicated that rhApoE/PTX/Aβ-CN-PMs treatment notably induced cell apoptosis compared to other groups, which might be attributed to the higher uptake of rhApoE/PTX/Aβ-CN-PMs on C6 cells and induced the apoptosis of tumor cells more effectively.

### Cell wound-healing assay

In vitro cell wound-healing assays have been widely used to determine cellular migration by analyzing changes in the width of scratches made in cell monolayers [46]. As shown in Fig. 8D, C6 cells in the wounded area in the control group gradually healed, and reduced cytotoxicity and inhibition of migration were observed after incubation with taxol, PTX/PMs, and PTX/Aβ-CN-PMs. However, the scratches in the rhApoE/PTX/Aβ-CN-PMs group remained clearly visible due to the greater cytotoxicity and distinct inhibition of migration of C6 cells compared to other groups, further confirming the results of the cellular uptake and antiproliferation assays. Hence, PTX/Aβ-CN-PMs formed the ApoE-enriched PC and had great potential for glioma treatment.

### Tumor-targeting efficacy of Aβ-CN-PMs on HUVECs and C6 cells co-cultured BBTB model

**Fig. 9:**
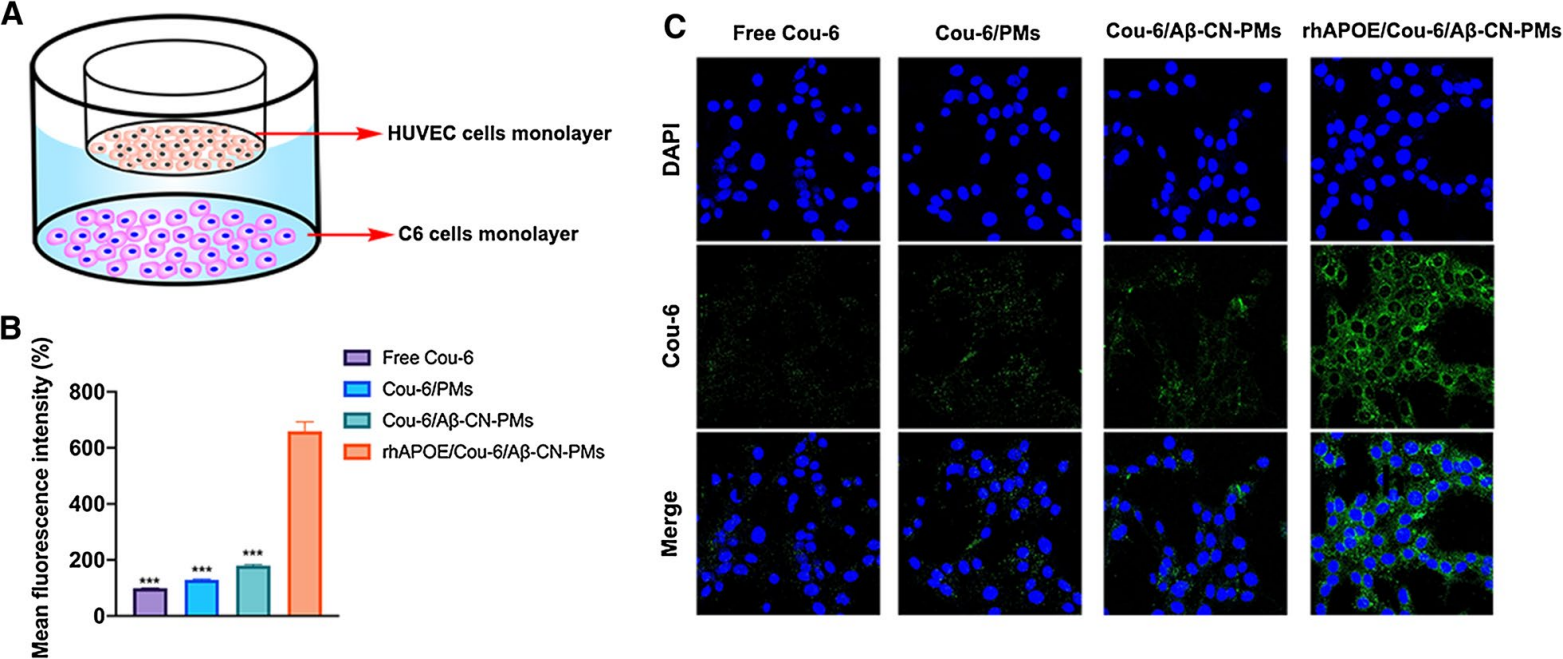
**A** Schematic diagram of the in vitro BBTB model established by co-cultured HUVECs and C6 cells into upper and lower chamber. **B** Quantitative analysis of C6 cells uptake in the lower chamber by flow cytometry after incubation with free Cou-6, Cou-6/PMs, Cou-6/Aβ-CN-PMs, rhApoE/Cou-6/Aβ-CN-PMs for 3 h, respectively. Means ± SD, n = 3; *P < 0.05, **P < 0.01, ***P < 0.001 versus the rhApoE/Cou-6/Aβ-CN-PMs group. **C** CLSM analysis of uptake by C6 cells in the lower chamber for 3 h incubation with free Cou-6, Cou-6/PMs, Cou-6/Aβ-CN-PMs and rhApoE/Cou-6/Aβ-CN-PMs in the upper chamber (magnification × 200)

A BBTB model was established through the co-culture of HUVECs and C6 cells into the upper and lower chambers, respectively. The BBTB-targeting and penetration ability of rhApoE/Cou-6/Aβ-CN-PMs was studied in a Transwell system using a co-culture non-contact BBTB model of HUVECs and C6 cells (Fig. 9A). Cou-6 was used as an indicator and was encapsulated to prepare Cou-6/PMs, Cou-6/Aβ-CN-PMs, and rhApoE/Cou-6/Aβ-CN-PMs. The cellular uptake of the different PMs on C6 cells in the lower compartment was measured using CLSM and flow cytometry. As shown in Fig. 9B and C, consistent with the cellular uptake data, the fluorescence signals in the rhApoE/Cou-6/Aβ-CN-PMs group were more intense and deeper than those of the free Cou-6, Cou-6/PMs, and Cou-6/Aβ-CN-PMs groups after 3 h incubation, indicating that rhApoE/Cou-6/Aβ-CN-PMs had an improved ability to cross the BBTB and target glioma cells through interacting with ApoE. For quantitative analysis, the results showed that the fluorescence intensity of Cou-6 in the rhApoE/Cou-6/Aβ-CN-PMs group was 6.63, 5.10, and 3.67-fold higher than that of Cou-6 in the free Cou-6, Cou-6/PMs, and Cou-6/Aβ-CN-PMs groups. These results indicated that the brain-targeting ApoE-enriched PC increased the transcytosis efficiency of rhApoE/Cou-6/Aβ-CN-PMs in an in vitro BBTB model. Therefore, the Aβ-CN peptide may be used as an excellent ligand for the formation of brain-targeting PCs.

### Biodistribution of micelles estimated using in vivo imaging

Based on the results of the transport capability across the BBTB, the rhApoE/Cou-6/Aβ-CN-PMs crossed the in vitro BBTB model and further efficiently targeted glioma cells. Therefore, the in vivo fluorescence signals in the brain and various organs were observed at 1, 2, 4, 24 h, and 36 h after the administration of 1,1-dioctadecyltetramethyl indotricarbocyanine iodide (DiR)/PMs and DiR/Aβ-CN-PMs to study the biodistribution of PMs and to confirm that the Aβ-CN peptide-modified PMs effectively form the ApoE-enriched PC with brain-targeting functions during in vivo circulation.

**Fig. 10:**
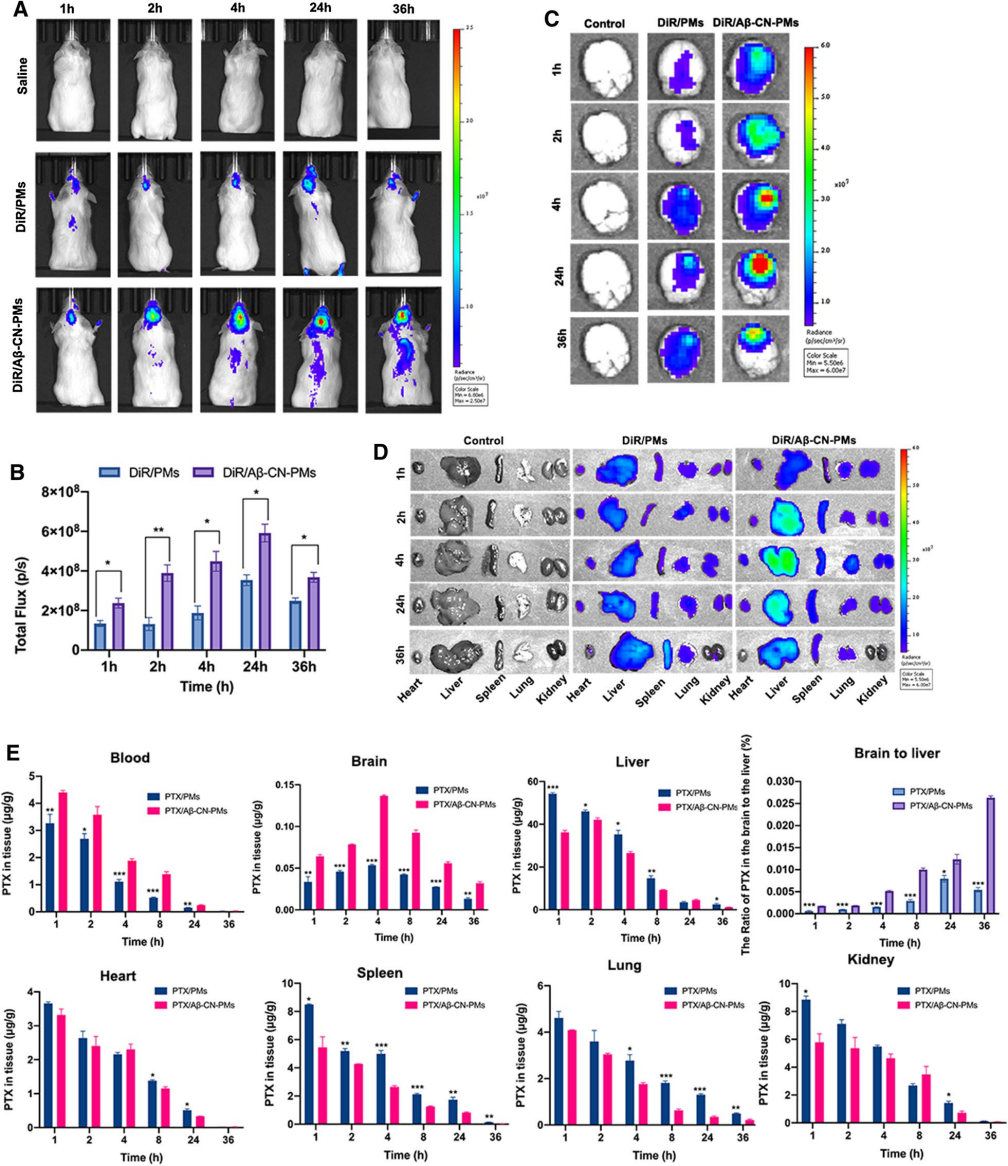
**A** In vivo fluorescence imaging of orthotopic glioma-bearing mice treated with saline, DiR/PMs and DiR/Aβ-CN-PMs at 1 h, 2 h, 4 h, 24 h, and 36 h. **B** In vivo radiant efficiency of orthotopic glioma-bearing mice at 1 h, 2 h, 4 h, 24 h, and 36 h. Means ± SD, n = 3; *P < 0.05, **P < 0.01, ***P < 0.001 versus the DiR/Aβ-CN-PMs group. **C** Ex vivo imaging of brains from orthotopic glioma-bearing mice, which were administrated with saline, DiR/PMs and DiR/Aβ-CN-PMs at 1 h, 2 h, 4 h, 24 h, and 36 h. **D** Ex vivo fluorescence imaging of the major organs (hearts, livers, spleens, lungs and kidneys) from orthotopic glioma mice after the administration with saline, DiR/PMs and DiR/Aβ-CN-PMs at 1 h, 2 h, 4 h, 24 h, and 36 h. **E** Biodistribution of PTX/PMs and PTX/Aβ-CN-PMs in mice at 1 h, 2 h, 4 h, 8 h, 24 h, and 36 h after intravenous injection (n = 3 for each group at each time point). Means ± SD, n = 3; *P < 0.05, **P < 0.01, ***P < 0.001 versus the PTX/Aβ-CN-PMs group

As illustrated in Fig. 10A, the fluorescence intensity in the brains of the DiR/PM group showed a mild increase with an increase in time and reached its peak at 24 h, then the fluorescence intensity in the brains started to decrease in both groups. Importantly, the fluorescence intensity in the brains of the mice in the DiR/Aβ-CN-PMs group was remarkably stronger than that in the DiR/PMs treatment group at any given time point, other images have been put in Additional file 1: Fig. S1. According to the statistical results in Fig. 10B, the fluorescence intensity in the brains of the mice in the DiR/Aβ-CN-PMs group were 1.77, 2.95, 2.39, 1.67, and 1.47-fold higher than those in the DiR/PMs group at 1, 2, 4, 24 h, and 36 h, respectively. The modification of PMs with the Aβ-CN peptide effectively formed ApoE-enriched PC to achieve the brain glioma-targeted delivery and prolong the circulation time in vivo compared to the DiR/PMs without Aβ-CN peptide modification. Meanwhile, ex vivo imaging of the brains confirmed the highest fluorescence intensity at the glioma site in the DiR/Aβ-CN-PMs group (Fig. 10C). As for other major organs, ex vivo fluorescence signals in both the DiR/PMs and DiR/Aβ-CN-PMs groups were observed (Fig. 10D); however, they were primarily distributed in the mononuclear phagocyte system-related organs, such as the liver and spleen [47, 48]. These results indicated that the formation of the ApoE-enriched PC on the Aβ-CN-PMs accelerated the rate of penetration across the BBB and BBTB, further selectively targeting the glioma regions through specific binding with LDLr and LRP1r expressed on the BBB and glioma cells.

### Tissue biodistribution

PTX distribution in tissue and blood was assessed in ICR mice following intravenous administration of PTX/PMs and PTX/Aβ-CN-PMs. Figure 10E presents the drug distribution of the two preparations in various tissues and blood. First of all, the PTX concentration in brains treated with PTX/Aβ-CN-PMs was obviously higher than that of PTX/PMs within 36 h and exhibited the peak at 4 h after injection. According to the statistical results, the concentration of PTX in the brains of PTX/Aβ-CN-PMs were 2.09, 1.70, 2.55, 2.19, 2.02 and 2.46-fold higher than that of PTX/PMs at 1, 2, 4, 8, 24, 36 h, respectively. Furthermore, the PTX concentration of PTX/Aβ-CN-PMs group was lower than that of PTX/PMs group in liver and spleen. In contrast, the PTX concentration in blood of PTX/Aβ-CN-PMs was higher than that of PTX/PMs at each time point from 1 to 36 h following administration. The reasons for the above results may be that the adsorbed ApoE on the surface of PTX/Aβ-CN-PMs is one of the dysopsonins and the ApoE-enriched protein corona protects the PTX/Aβ-CN-PMs, prevents their phagocytosis and extends circulation time of PTX/Aβ-CN-PMs in vivo. While the differences in PTX concentration of heart, lung and kidney between PTX/PMs and PTX/Aβ-CN-PMs were not significant at the desired time points and the drug concentration showed a decreasing pattern from 1 to 36 h following administration. To evaluate the targeting efficiency of PTX/Aβ-CN-PMs, the ratio of drug concentration in the brain to the liver was calculated. The results in Fig. 10E showed that the brain-to-liver ratio in the PTX/Aβ-CN-PMs group was stronger than that in the PTX/PMs group and it was increasing with time. These results directly indicated that Aβ-CN peptide-modified PTX-loaded PMs possessed a desirable biodistribution profile, suggesting that ApoE-enriched PC effectively mediated brain-targeting drug delivery system.

### In vivo glioma brain biodistribution

**Fig. 11:**
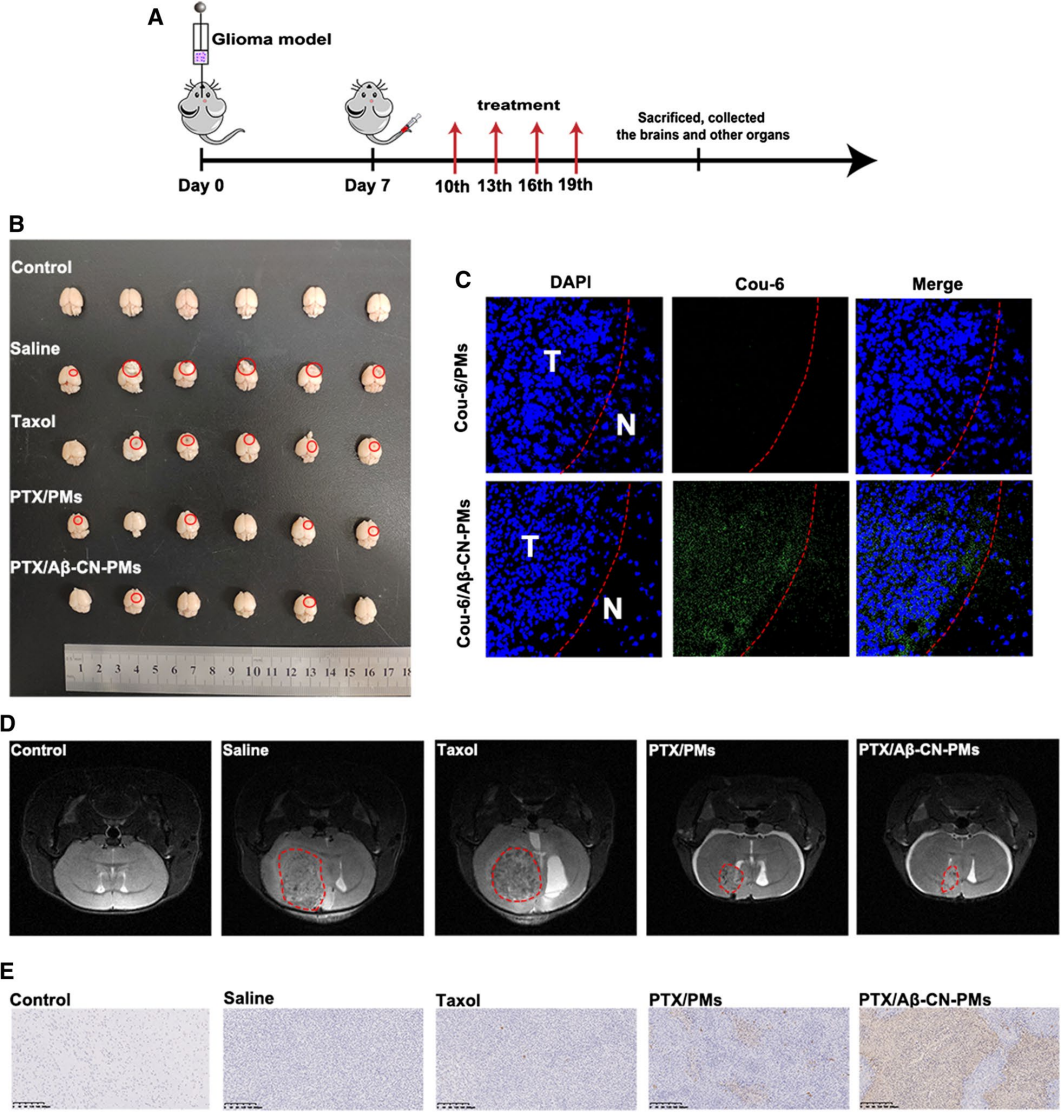
**A** Schematic diagram of in vivo anti-glioma effect study **B** Images of brain tissues isolated from orthotopic glioma mice after treatment with saline, taxol, PTX/PMs, and PTX/Aβ-CN-PMs. **C** CLSM images of brain sections from orthotopic glioma mice of Cou-6/PMs and Cou-6/Aβ-CN-PMs. Blue, cell nuclei stained with DAPI; Green, Cou-6/PMs and Cou-6/Aβ-CN-PMs; N, normal brain sections; T, glioma section; Red line, boundary of the glioma (magnification × 200). **D** MRI of brain in control group and brains from orthotopic glioma mice after treatment with saline, taxol, PTX/PMs, and PTX/Aβ-CN-PMs. **E** TUNEL assay of orthotopic glioma tumor tissues isolated from mice treated with saline, taxol, PTX/PMs, and PTX/Aβ-CN-PMs observed by optical microscope (magnification × 100). Scale bar = 200 μm. Brown areas showed apoptosis of tumor cells

To clarify the distribution of PMs in glioma-bearing brains, frozen sections were prepared, and the nuclei were stained with DAPI after 3 h post-injection of Cou-6/PMs and Cou-6/Aβ-CN-PMs, which were observed and studied qualitatively using CLSM. As shown in Fig. 11C, glioma tissues were much denser than normal brain tissues due to the rapid proliferation of glioma cells. They showed obvious boundaries between glioma regions and normal tissues. Weaker fluorescence signals were observed in the glioma regions of the glioma-bearing mice treated with Cou-6/PMs; however, stronger fluorescence signals accumulated in the tumor after treatment with Cou-6/Aβ-CN-PMs, which was consistent with the findings of the cellular uptake study and the in vivo biodistribution experiment [49, 50]. These results demonstrated that the Aβ-CN peptide specifically and efficiently captured ApoE in vivo and that the ApoE-enriched PC effectively targeted the LDLr and LRP1r on the BBB and glioma cells, significantly enhancing the drug penetration in tumor tissue. PTX/Aβ-CN-PMs had great potential to be a target-specific drug delivery system for glioma or other brain diseases.

### Evaluation of anti-glioma effects in vivo

Glioma-bearing mouse models were established as a method to evaluate the anti-glioma effects of the different formulations. The mice were randomly divided into four groups (n = 6): saline, taxol, PTX/PMs, and PTX/Aβ-CN-PMs. The mice were sacrificed on the third day after the final injection to collect the brain tissues and other organs (hearts, livers, spleens, lungs, and kidneys) (Fig. 11A). The anti-glioma effects were evaluated using magnetic resonance imaging (MRI), and the results are shown in Fig. 11D. It is not difficult to confirm that the modification of PMs with the Aβ-CN peptide further improved the anti-glioma effects, and mice treated with PTX/Aβ-CN-PMs exhibited the smallest tumor volume (red circles) among all groups. The anti-glioma effect of PTX/Aβ-CN-PMs was better than that of the other formulations, consistent with the results shown in Fig. 11B. As shown in Fig. 11B, compared with brain tissues in the PTX/Aβ-CN-PMs group, glioma tissues were distinctly observed in the saline, taxol, and PTX/PMs groups, indicating that taxol and PTX/PMs had no notable inhibitory effect on glioma progression. These results suggested that the PTX/Aβ-CN-PMs delivery system facilitated the anti-glioma treatment efficacy of PTX in vivo.

To further investigate the apoptosis of tumor cells in brain tissues after treatment with the different formulations, brain paraffin sections were obtained and analyzed via terminal deoxynucleotidyl transferase-dUTP nick end labeling (TUNEL) staining. As shown in Fig. 11E, compared with saline, taxol, and PTX/PMs, PTX/Aβ-CN-PMs induced more significant apoptosis in glioma cells, which suggested that PTX/Aβ-CN-PMs penetrated deeper into glioma tissues and killed more tumor cells than taxol and PTX/PMs, thus inhibiting the growth of glioma. This may be due to the specific binding of the ApoE-enriched protein corona absorbed on PTX/Aβ-CN-PMs to overexpressed LRP1r/LDLr on the BBB and glioma cells, leading to more accumulation of PTX at glioma sites rather than other locations in the brain. Meanwhile, these results are consistent with the previous reports and further suggests that the adsorbed the ApoE-enriched protein corona on PTX/Aβ-CN-PMs effectively achieve brain-targeted drug delivery through the LDLr- and LRP1r-mediated brain delivery mechanism [51–56]. Thus, PTX/Aβ-CN-PMs with ApoE-enriched protein corona not only improve the efficacy but also increase the safety of PTX/Aβ-CN-PMs in the brain.

**Fig. 12:**
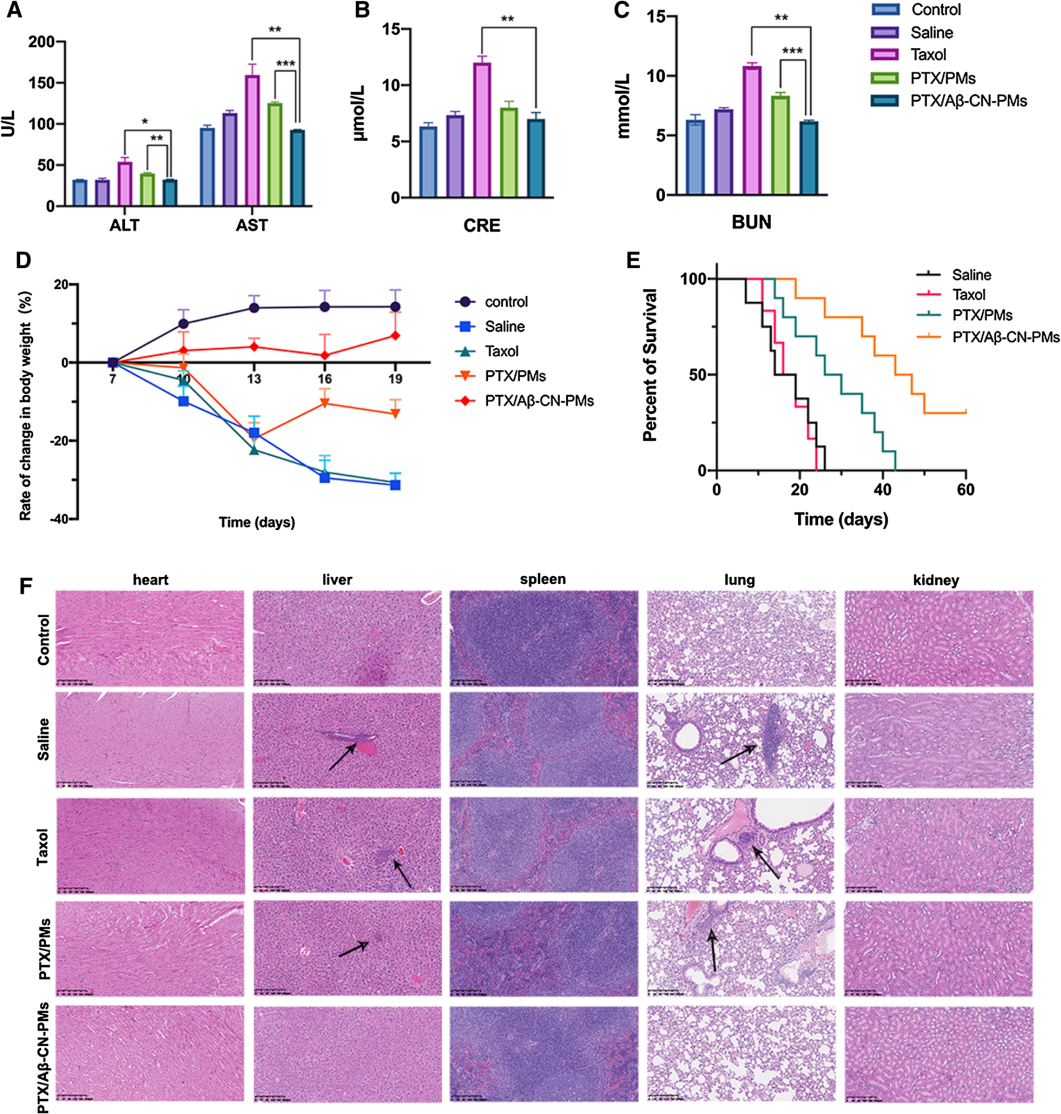
In vivo safety assessment and Histological examination analysis. **A**–**C** AST, ALT, CRE, and BUN from the blood samples isolated from orthotopic glioma tumor tissues after treatment with saline, taxol, PTX/PMs, and PTX/Aβ-CN-PMs, respectively, Means ± SD, n = 6; *P < 0.05, **P < 0.01, ***P < 0.001 versus the PTX/Aβ-CN-PMs group. **D** The rate of change in body weight of orthotopic glioma tumor mice in the different groups at different time points (n = 6). **E** Kaplan–Meier survival curves of percentage survival of orthotopic glioma mice treated with saline, taxol, PTX/PMs, and PTX/Aβ-CN-PMs, respectively (n = 10). **F** H&E staining of hearts, livers, spleens, lungs and kidneys after treatment with saline, taxol, PTX/PMs, and PTX/Aβ-CN-PMs observed by optical microscope (magnification × 100). Scale bar = 200 μm

Kaplan–Meier survival curves showed that the median survival time of the mice treated with PTX/Aβ-CN-PMs was 45 days, which was significantly longer than that of the mice treated with saline (16.5 days), taxol (17.5 days), and PTX/PMs (28 days) (Fig. 12E). This demonstrated that the injection of PTX/Aβ-CN-PMs could effectively prolong the survival time of glioma-bearing mice. These results also indicated that Aβ-CN peptide-modified PTX-loaded PMs exhibited a significant improvement in glioma treatment compared with free PTX and PTX/PMs.

To evaluate the anti-metastatic capacity of PTX/Aβ-CN-PMs, the major organs (hearts, livers, spleens, lungs, and kidneys) were harvested three days after the final injection, sections of these organs were prepared, and were stained with hematoxylin and eosin (H&E). As shown in Fig. 12F, the pathological images of the hearts, spleens, and kidneys in all groups had no obvious metastases and no apparent inflammatory response or damage, indicating that all formulations had good safety and biocompatibility. In addition, a metastatic region was observed in the lungs and livers in the saline, taxol, and PTX/PMs groups (red circles); however, no distinguishable tumor micrometastasis was found in the PTX/Aβ-CN-PMs group, suggesting that PTX/Aβ-CN-PMs had a greater ability to inhibit glioma metastasis than taxol or PTX/PMs.

### Safety assessment

The in vivo safety of PTX/Aβ-CN-PMs was evaluated based on changes in mouse body weight and hematological analysis. The body weight change reflected the progress of glioma proliferation and systemic toxicity of the formulations to some extent. In order to provide a more accurate description of weight change, the rate of change in body weight curve was exhibited in Fig. 12D, the results showed that the mice treated with PTX/Aβ-CN-PMs showed a positive trend change in body weight, while the rate of change in body weight in the other groups (saline, taxol, and PTX/PMs group) was negative. That is, the weight of mice in the PTX/Aβ-CN-PMs group increased, in contrast, apparent weight loss was observed in the saline and taxol groups. This phenomenon may be caused by the systemic toxicity and side effects of taxol. The results of the hematological analysis (Fig. 12A–C) showed that the levels of alanine aminotransferase (ALT), aspartate aminotransferase (AST), creatinine (CRE), and blood urea nitrogen (BUN) in the mice in the taxol group remarkably improved, while those in the mice treated with PTX/Aβ-CN-PMs were relatively stable and were consistent with those in the control group. These above results indicated that PTX/Aβ-CN-PMs might contribute to reducing the toxicity and adverse effects of PTX and improving the safety of chemotherapeutic drugs.

However, the understanding of the control and mechanism of protein corona formation needs to be further enhanced. In the future research, we will develop more analytical techniques to characterize nanoparticle‒protein interactions for a comprehensive understanding, which will be beneficial to control and exploit the nanoparticle‒protein interactions for targeted drug delivery.

## Conclusions

In summary, we developed an ApoE-enriched PC-mediated brain-targeted drug delivery system for glioma treatment. The Aβ-CN peptide was used as a ligand for the formation of the ApoE-enriched PC with brain-targeting properties. The ApoE PC played a major role in targeting LDLr and LRP1r on the BBB and glioma cells. As shown by the in vivo and in vitro results, PMs modified with the Aβ-CN peptide bound to ApoE efficiently and accumulated rapidly in the tumor tissues, which significantly improved cellular uptake efficiency on C6 cells and HUVECs and the rate of transport across the BBB and BBTB. More importantly, PTX/Aβ-CN-PMs demonstrated significant inhibitory effects on cell proliferation, tumor growth, and metastasis and prolonged the survival time of glioma-bearing mice without obvious systemic toxicity. These findings indicate that we should regard nanoparticle‒protein interactions as an opportunity to design rational nanocarriers that will be more suitable for targeted drug delivery instead of considering them an obstacle. The regulation of specific PCs in situ might provide more insights and better chances for PC-mediated systemic drug delivery.

## Materials and methods

### Materials, cells and animals

Aβ_25–35_, with the amide form of the C-terminal methionine (Aβ-CN), was purchased from GL Biochem Ltd. (Shanghai, China). mPEG_2000_-NH_2_ and Mal-PEG_2000_-NH_2_ were purchased from Tanshtech Ltd. (Guangzhou, China). Dicyclohexylcarbon diamine (DCC), 4-dimethylaminopyridine (DMAP), and *N*-hydroxysuccinimide (NHS) were obtained from Inno-chem Ltd. (Beijing, China). PTX was purchased from Dalian Meilun Biotechnology Co., Ltd. (Dalian, China). rhApoE was purchased from Solarbio Science & Technology Co., Ltd. (Beijing, China). Acetonitrile, methanol, and Cou-6 (high-performance liquid chromatography (HPLC) grade) were purchased from Sigma-Aldrich Co. (St. Louis, MO, USA). DiR was obtained from Biotium Inc. (Hayward, CA, USA). The Annexin V-FITC/PI Apoptosis Detection kit and CCK-8 were purchased from Dojindo Laboratories (Kumamoto, Japan). Hoechst 33258 and DAPI were purchased from Solarbio Science & Technology Co., Ltd. Fetal bovine serum (FBS) and 0.25% trypsin were obtained from Thermo Fisher Scientific Co., Ltd. (Beijing, China). Dulbecco’s Modified Eagle medium (DMEM), Roswell Park Memorial Institute-1640 (RPMI-1640) medium, and PBS were purchased from GE Healthcare (Boston, MA, USA). Hank’s balanced salt solution was purchased from Thermo Fisher Scientific (Waltham, MA, USA). All other chemicals were of analytical purity and were used without further purification.

HUVECs and C6 cells were purchased from BMCR (China). HUVEC lines were cultured in DMEM with 10% FBS and maintained in a humidified atmosphere containing 5% CO_2_ at 37 °C. C6 cells were cultured in RPMI-1640 medium supplemented with 10% FBS at 37 °C in an atmosphere of 5% CO_2_. All cell experiments were performed on cells in the logarithmic stage of growth.

Male Institute of Cancer Research (ICR) mice (18–20 g) were obtained from Vital River Laboratory Animal Technology Co., Ltd. (Beijing, PR China) and maintained under standard housing conditions. All animal experiments were conducted in accordance with the guidelines prepared and approved by the Laboratory Animal Ethics Committee of the Institute of Materia Medica in CAMS and PUMC.

### Synthesis and characterization of PEG–PLA and Aβ-CN-PEG-PLA materials

#### Synthesis and characterization of mPEG–PLA and Mal-PEG–PLA

mPEG–PLA was prepared by conjugating PLA_1300_-COOH with mPEG_2000_-NH_2_ through an amidation reaction. First, PLA_1300_-COOH (Mw 1300, 300 mg, 0.23 µmol), DMAP (5.60 mg, 0.046 µmol), DCC (57.13 mg, 0.27 µmol), and NHS (31.87 mg, 0.27 µmol) were dissolved in 5 mL of anhydrous CH_2_Cl_2_ in a 50 mL round-bottomed flask under stirring, which was protected in a nitrogen atmosphere. After a 1 h reaction, the 1,3-dicyclohexyl urea by-product was removed via filtration. Next, mPEG_2000_-NH_2_ (MW 2000, 553.8 mg, 0.27 µmol) was added, and the reaction was stirred for a further 48 h at room temperature under a nitrogen atmosphere. After completion of the reaction, the reaction mixture was filtered and precipitated in cold methyl tertiary-butyl ether. Unreacted mPEG_2000_-NH_2_ was then removed by dialyzing against distilled water for 24 h (MWCO 3.5 kDa). The purified mPEG_2000_–PLA_1300_ was obtained via freeze-drying for further experiments. The Mal-PEG_2000_–PLA_1300_ copolymer was prepared using a similar method, except for mPEG_2000_-NH_2_. The structures of the mPEG_2000_–PLA_1300_ and Mal-PEG_2000_–PLA_1300_ copolymers were characterized via ^1^H-NMR spectroscopy (400 MHz, Varian Medical Systems, Inc., Palo Alto, CA, USA) [27, 28].

### Synthesis and characterization of Aβ-CN-PEG–PLA

To synthesize the Aβ-CN-modified targeted carrier, the Aβ-CN peptide with an additional cysteine in its N-terminus was coupled to the terminal mal groups of Mal-PEG_2000_–PLA_1300_ using the Michael addition reaction, as described in the previous section [29–31]. First, Mal-PEG_2000_–PLA_1300_ was mixed with an aqueous solution of Aβ-CN to form a suspension. A 4-(2-hydroxyethyl)-1-piperazineethanesulfonic acid buffer (HEPEs, 0.1 M, pH 8.0) was then added to the suspension, and the reaction was stirred using a magnetic stirrer for 24 h under a nitrogen environment at room temperature. The unreacted Aβ-CN peptide was removed via dialysis (MWCO 3.5 kDa) against distilled water for 24 h. Pure Aβ-CN- PEG_2000_–PLA_1300_ was obtained by lyophilization. The Aβ-CN peptide coupled to Mal-PEG_2000_–PLA_1300_ was identified using ^1^H-NMR spectroscopy (400 MHz, Varian Medical Systems, Inc., Palo Alto, CA, USA).

### Preparation and characterization of PTX-loaded Aβ-CN-PEG–PLA micelles

PTX-loaded Aβ-CN-PEG_2000_–PLA_1300_ micelles (PTX/Aβ-CN-PMs) were prepared using the film hydration method as reported previously with minor changes. Briefly, 20 mg of mPEG_2000_–PLA_1300_, 1.8 mg of Aβ-CN-PEG_2000_–PLA_1300_, and 2 mg of PTX were co-dissolved in 5 mL of acetonitrile, and the mixture was then sonicated for 3 min for further dissolution. The solution was rotary evaporated at 45 °C to form a thin film, followed by hydration with 4 mL of PBS (pH 7.4). The obtained micelle solution was allowed to stand for 30 min to fully self-assemble and then sonicated in an ice bath for 10 min. For the preparation of PTX-loaded mPEG_2000_–PLA_1300_ micelles (PTX/PMs), a similar procedure was performed, except for Aβ-CN-PEG_2000_–PLA_1300_. Dynamic light scattering and TEM were performed to characterize the particle size, zeta potential, morphology, and stability of the micelles. The EE% and LC% of PTX in PTX/Aβ-CN-PMs were quantified using HPLC (Agilent 1200, Agilent Technologies, Santa Clara, CA, USA) with a C18 column and were calculated as follows [32–34]:

$${\text{EE}}\% = \frac{\text{Weight of encapsulated drug}}{\text{Weight of total drug}} \times 100\%$$

$${\text{DL}}\% = \frac{{\text{Weight of encapsulated drug}}}{{\text{Weight of total drug and polymer}}} \times 100\%$$

To prepare Cou-6 or DiR-labeled PMs, the PTX in PTX/Aβ-CN-PMs and PTX/PMs was replaced by Cou-6 or DiR. Other steps were implemented similar to those described above.

### Interaction of ApoE on Aβ-CN-PMs in vitro

To investigate the binding between ApoE and Aβ-CN-PMs, whole blood samples were acquired from ICR mice and stored in tubes containing heparin sodium. Plasma samples were prepared via centrifugation at 3000 rpm for 10 min, and the supernatant was collected for further experiments. PTX/Aβ-CN-PMs or PTX/PMs (200 μL) in PBS were incubated with the same volume of plasma at 37 °C for 1 h with gentle shaking. After incubation, the mixtures were centrifuged at 14,000 rpm for 1 h at 4 °C, and the pellet was washed with cold PBS thrice to remove unbound or loosely bound proteins. The ApoE inside the hard PC of the pellet was detected via western blotting analysis [17, 19, 35].

### Characterization of protein corona

Mouse whole blood was collected from ICR mice and mixed with heparin sodium. Mouse plasma was prepared by centrifugation at 3000 rpm for 10 min and stored at − 80 °C for further experiments. Plasma sample was thawed and warmed at room temperature before use. PTX/Aβ-CN-PMs or PTX/PMs in PBS was incubated with equal volume of plasma at 37 °C for 1 h, and the mixture was centrifuged at14000 rpm for 1 h to pellet the nanoparticle–protein complex. The pellet was washed with cold PBS thrice to remove unbound or loosely bound proteins. The enzymatic digestion of the proteins on the nanoparticles surface was performed. Samples were destined, reduced, and alkylated, followed by trypsin digestion. The digested peptides were extracted, resuspended in a solution (0.1% formic acid and 2% acetonitrile), and analyzed by LC − MS/MS. Mass spectrometry data were used to identify proteins from the protein database using Maxquant (1.6.2.10).

### In vitro release study

The in vitro release profile of PTX was studied in a sodium salicylate solution (1 M, pH 7.4) using the dialysis diffusion method. One milliliter of taxol, PTX/Aβ-CN-PMs, and rhApoE/PTX/Aβ-CN-PMs at a PTX concentration of 1 mg/mL were added to dialysis bags (MWCO, 12 kDa). The bags were immersed in 30 mL of sodium salicylate solution (1 M, pH 7.4) and shaken at 100 rpm at 37 °C. Samples (0.5 mL) were obtained at each time interval, and an equal amount of fresh release medium was added immediately. The collected samples were centrifuged (12,000 rpm, 10 min), and the supernatant was analyzed using a previously established HPLC method to detect the content of PTX. Finally, the cumulative release of PTX was calculated, and an in vitro release curve was plotted.

### In vitro cytotoxicity

The cytotoxicity of blank Aβ-CN-PMs and blank rhApoE/Aβ-CN-PMs against C6 cells and HUVECs was measured using a CCK-8 assay. Briefly, cells were seeded in a 96-well plate at a density of 5000 cells per well and incubated at 37 °C in a humidified atmosphere with 5% CO_2_ for 24 h. Subsequently, the spent culture media was removed and replaced with different concentrations of blank Aβ-CN-PMs and blank rhApoE/Aβ-CN-PMs at equivalent concentrations of PTX, ranging from 0.001 μg/mL to 10 μg/mL. After the desired incubation time, the micellar solution was replaced with CCK-8 solution, and the cells were cultured in CCK-8 solution at 37 °C for 3 h. The optical density (OD) was measured at 450 nm using a Synergy H1 microplate reader (BioTek, Dallas, TX, USA). Cells treated with complete culture medium were used as controls. Each group comprised three samples in parallel, and the cell viability was calculated as follows:

$$\mathrm{Cell viability }(\mathrm{\%}) =\frac{\mathrm{ODtest}-\mathrm{ODblank}}{\mathrm{OD control}-\mathrm{ODblank}} \times 100\mathrm{\%}$$

Furthermore, the growth inhibitory effects of taxol, PTX/Aβ-CN-PMs, and rhApoE/PTX/Aβ-CN-PMs on C6 cells were also studied using a CCK-8 assay. C6 cells were seeded in a 96-well plate at the same concentration and incubated as described above. The spent culture medium was then replaced with different concentration of taxol, PTX/Aβ-CN-PMs, and rhApoE/PTX/Aβ-CN-PMs at PTX concentration ranging from 0.001 to 10 μg/mL followed by incubation for 24 h or 48 h. Subsequently, the cells were treated with CCK-8 solution at 37 °C for 3 h according to the manufacturer’s instructions. Cells treated with complete culture medium were used as controls. Cell viability was calculated using the same experimental steps as described above.

### Cellular uptake assays

Cou-6 replaced PTX in PTX/Aβ-CN-PMs to observe and analyze the cellular uptake of the different formulations. HUVECs and C6 cells were seeded at a density of 1.5 × 10^4^ cells per well in 12-well plates and incubated for 24 h. The cells were then cultured with free Cou-6, Cou-6/PMs, Cou-6/Aβ-CN-PMs, and rhApoE/Cou-6/Aβ-CN-PMs (Cou-6 concentration of 1 µg/mL) for 2 h. Cells were treated with serum-free medium only as a control. For qualitative analysis, the cells were washed thrice with cold PBS, fixed with 4% paraformaldehyde solution for 15 min, washed thrice, stained with DAPI for 10 min, and finally observed via CLSM (Carl Zeiss LSM 710; Carl Zeiss Microscopy, Jena, Germany). For quantitative analysis, the cells were washed thrice with cold PBS, and 0.25% trypsin was added. After a 2 min incubation at 37 °C, trypsin was inactivated by adding 1 mL of culture medium containing 10% FBS. The cells were then centrifuged (500×*g*, 5 min) and the supernatant was removed. The cells were then resuspended in 300 μL of PBS, and the fluorescence intensity was determined using flow cytometry using the FACS Calibur flow cytometer (Becton Dickinson, Franklin Lakes, NJ, USA).

To study the time dependence of cellular uptake in C6 cells and HUVECs, cells were incubated with rhApoE/Cou-6/Aβ-CN-PMs (Cou-6 concentration of 1 µg/mL) for 15 min, 30 min, 1 h, and 2 h. The procedures described above were performed to estimate the cellular uptake efficiency using flow cytometry and CLSM.

To further confirm the cellular uptake mechanism of rhApoE/Cou-6/Aβ-CN-PMs, C6 cells were incubated with various endocytic inhibitors, including chlorpromazine (10 μg/mL), methyl-β-cyclodextrin (M-β-CD, 5 mg/mL), NaN_3_ (3 mg/mL), colchicine (4 μg/mL), chloroquine (5 μg/mL), amiloride (12.5 μg/mL), brefeldin A (25 μg/mL), and genistein (27 μg/mL) for 1 h, followed by incubation with rhApoE/Cou-6/Aβ-CN-PMs for 2 h. Subsequently, the steps described above were implemented to evaluate the fluorescence intensity using flow cytometry.

### Tumor-targeting efficacy of Aβ-CN-PMs in a HUVEC/C6 co-culture BBTB model

To evaluate the BBTB crossing and targeting ability, a HUVEC/C6 co-culture BBTB in vitro model was established as previously described. Prior to use, 12-well Transwell plates with a pore size of 0.4 μm were coated with 0.5% gelatin/PBS (v/v) for 1 h. Subsequently, the gelatin solution was removed, and 0.5 mL of HUVEC suspension at a cell density of 2 × 10^5^ per well was seeded onto the apical Transwell chamber. When the transendothelial electrical resistance was > 200 Ω cm^2^, 1.5 mL of C6 cells at a density of 1 × 10^5^ per well were seeded onto the lower chamber of the Transwell plate. Two days later, the apical culture medium was replaced and HUVECs were incubated with free Cou-6, Cou-6/PMs, Cou-6/Aβ-CN-PMs, and rhApoE/Cou-6/Aβ-CN-PMs for 3 h. After incubation, the C6 cells in the lower chamber were washed with cold PBS thrice and fixed with 4% paraformaldehyde for 15 min. The C6 cell nuclei were then stained with DAPI, and the BBTB penetration and targeting ability of Aβ-CN-PMs were analyzed using CLSM [3, 29, 36]. For quantitative analysis, the fluorescence intensity of the C6 cells was determined through flow cytometry using the same experimental steps as described above in “Cellular uptake assays” section.

### Cell apoptosis assay

C6 cells were seeded into 12-well plates at a density of 1 × 10^5^ cells per well and cultured at 37 °C for 24 h. The cells were treated for 48 h with taxol, PTX/PMs, PTX/Aβ-CN-PMs, and rhApoE/PTX/Aβ-CN-PMs (at a PTX concentration of 1 μg/mL), with culture medium as a control. After incubation, for qualitative analysis, the cells were fixed with 4% paraformaldehyde at 37 °C for 15 min, stained with Hoechst 33258 at 37 °C for 10 min, and then washed with cold PBS thrice. Stained cells were examined using CLSM. For quantitative analysis, the apoptosis of C6 cells was analyzed using the Annexin V-FITC/PI double staining assay according to the Annexin V-FITC and PI staining instructions and evaluated using flow cytometry.

### Cell wound-healing assay

The inhibitory capacity of rhApoE/PTX/Aβ-CN-PMs on C6 cell migration was determined using a wound-healing assay. Briefly, C6 cells were plated onto 6-well plates at a density of 2 × 10^5^ cells per well and incubated until a confluent cell monolayer formed in each well (> 90% confluence). Wounds were created using a sterile 200 μL plastic pipette. The cell monolayers were then washed twice with PBS to remove cell fragments, and the wound edges at the bottom of the plates were observed. The cells were incubated with taxol, PTX/PMs, PTX/Aβ-CN-PMs, and rhApoE/PTX/Aβ-CN-PMs (at a PTX concentration of 1 μg/mL) in RPMI-1640 medium with 1% FBS at 37 °C. Photographs of the wounds were acquired using an inverted light microscope (Olympus, Hamburg, Germany) at 0, 12, 24, and 48 h.

### Biodistribution of Aβ-CN-PMs determined using in vivo imaging

An orthotopic glioma model was established by injecting C6 cells (2 × 10^5^ cells in 4 μL of PBS) into the right side of the brain of each male ICR mouse. Briefly, each mouse was anesthetized, and the skull was exposed by eliminating tissue from the skull surface with 5% hydrogen peroxide. A hole was drilled 1.8 mm lateral to the right of the sagittal suture to a depth of 3.0 mm, and the cells were seeded stereotactically into the targeted location. The scalp incision was sutured after sealing with bone wax. Mice in the control group were injected with normal saline as a substitute for C6 cells into the right side of the brain.

Seven days after implantation, the mice were divided randomly into three groups (n = 3) and injected with saline, DiR/PMs, and DiR/Aβ-CN-PMs at a dose of 10 μg/kg of DiR via the tail vein. In vivo fluorescence imaging post-injection was acquired at different time points (1 h, 2 h, 4 h, 24 h and 36 h) using an IVIS Spectrum CT in vivo imaging system (Caliper Life Sciences Inc., Mountain View, CA, USA). The mice were then sacrificed, and the brains and other major organs (hearts, livers, spleens, lungs, and kidneys) were harvested. Ex vivo imaging was performed to evaluate the biodistribution of DiR/Aβ-CN-PMs.

### Tissue biodistribution

Tissue biodistribution was performed on the healthy male ICR mice. The mice were randomly divided into two groups (n = 3). PTX/PMs, and PTX/Aβ-CN-PMs were administered via the tail vein at a dose of 7.5 mg/kg of PTX. At 1 h, 2 h, 4 h, 8 h, 24 h, and 36 h after the administration, these mice were sacrificed and their blood, hearts, livers, spleens, lungs, kidneys, and brains were taken. Blood and organs were rinsed with saline and then stored at − 80 °C until analysis. The organ samples were homogenized in fivefold volumes of saline. 100 μL homogenate or 50 μL plasma was pretreated via protein precipitation procedure using acetonitrile and the supernatant was analyzed by HPLC/MS using docetaxel as the internal standard.

### In vivo glioma-bearing brain biodistribution

ICR mice bearing glioma were established as described above. Seven days after glioma implantation, Cou-6/PMs and Cou-6/Aβ-CN-PMs were administered via the tail vein at a dose of 1.5 mg/kg of Cou-6. Three hours post-injection, the mice were anesthetized, followed by heart perfusion with saline and 4% paraformaldehyde. The brains of the mice were then collected, and frozen sections of 10 mm thickness were prepared. The nuclei were then stained with DAPI. After washing with PBS, the sections were observed using CLSM to explore the fluorescence distribution at the glioma site.

### Evaluation of anti-glioma effects in vivo

ICR mice bearing glioma were used to evaluate the anti-glioma effects of PTX/Aβ-CN-PMs. Seven days after glioma implantation, the mice were randomly classified into four groups (n = 6). Saline, taxol, PTX/PMs, and PTX/Aβ-CN-PMs were administered via the tail vein at a dose of 7.5 mg/kg of PTX at 7, 10, 13, 16, and 19 days after implantation. Meanwhile, the body weights of the mice were determined every three days. On the third day after the final injection, the mice were anesthetized, and the glioma were evaluated using MRI (PharmaScan 70 T/16, Bruker, US). Serum samples were collected, and serum biochemical indicators were analyzed to investigate the safety of PTX/Aβ-CN-PMs in vivo. Other tissues (hearts, livers, spleens, lungs, and kidneys) were harvested and treated with 4% formaldehyde tissue fixative for staining with H&E to assess the anti-metastatic effects of PTX/Aβ-CN-PMs. The TUNEL assay was performed to detect cell apoptosis in the glioma area.

### Survival analysis

To investigate the effects of different formulations on the survival time of tumor-bearing mice, glioma-bearing ICR mice were randomly divided into four groups (n = 10 each). The same administration procedure was performed as described in “Evaluation of anti-glioma effects in vivo” section. The survival times were recorded from glioma implantation to death, and the survival curves and statistical differences were analyzed using the Kaplan–Meier method.

### Statistical analysis

All data are presented as the mean ± standard deviation. Differences between any two groups were assessed using analysis of variance. Statistically significant differences were defined as *P < 0.05, **P < 0.01, and ***P < 0.001.

## Supplementary Information

**Additional file 1.** Biodistribution of micelles estimated using in vivo imaging.

## Data Availability

All data generated or analyzed during this study are included in this published article.
