# Supplementary material for: Novel brain-targeted nanomicelles for anti-glioma therapy mediated by the ApoE-enriched protein corona in vivo
Source: J Nanobiotechnology. 2021 Dec 28;19:453. doi: 10.1186/s12951-021-01097-8 (PMC8715648; doi:10.1186/s12951-021-01097-8)
Supplement: Supplementary file 1 — Additional file 1. Biodistribution of micelles estimated using in vivo imaging. [file 12951_2021_1097_MOESM1_ESM.docx]

**Novel brain-targeted nanomicelles for anti-glioma therapy mediated by the ApoE-enriched protein corona in vivo**

Zhe-Ao Zhang ^1,2^, Xin Xin^1,2^, Chao Liu^1,2^, Yan-hong Liu^1,2^, Hong-Xia Duan^1,2^, Ling-ling Qi^1,2^, Ying-Ying Zhang^1,2^, He-ming Zhao^1,2^, Li-Qing Chen^1,2^, Ming-Ji Jin^1,2^, Zhong-Gao Gao^1,2*^and Wei Huang^1,2*^

**Biodistribution of micelles estimated using in vivo imaging**


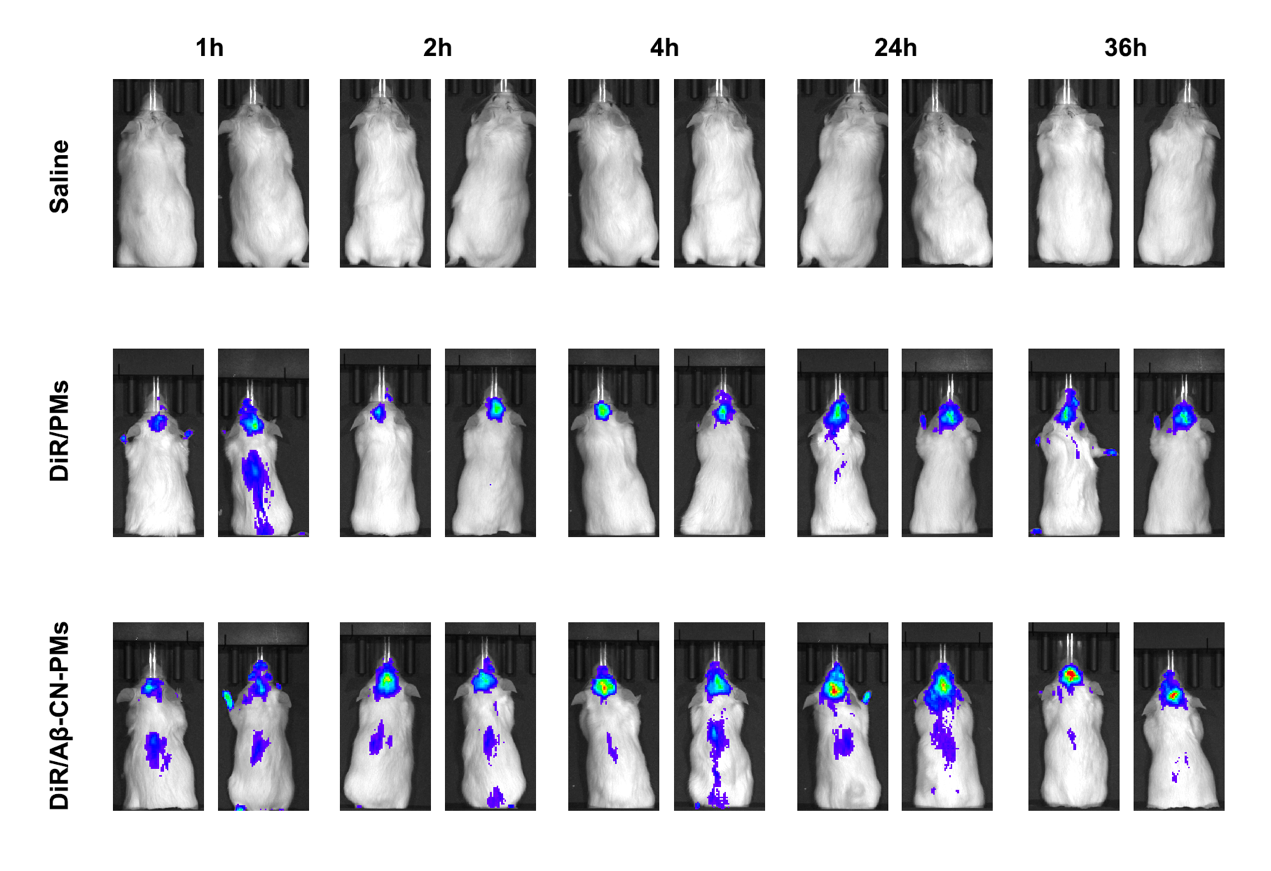


**Fig.S1.** *In vivo* fluorescence imaging of orthotopic glioma-bearing mice treated with saline, DiR/PMs and DiR/Aβ-CN-PMs at 1 h, 2 h, 4 h, 24 h, and 36h.
